# Fibril structures of TFG protein mutants validate the identification of TFG as a disease-related amyloid protein by the IMPAcT method

**DOI:** 10.1093/pnasnexus/pgad402

**Published:** 2023-11-20

**Authors:** Gregory M Rosenberg, Romany Abskharon, David R Boyer, Peng Ge, Michael R Sawaya, David S Eisenberg

**Affiliations:** Department of Chemistry and Biochemistry, UCLA-DOE Institute, Molecular Biology Institute, UCLA, Los Angeles, CA 90095, USA; Department of Biological Chemistry, UCLA-DOE Institute, Molecular Biology Institute, UCLA, Los Angeles, CA 90095, USA; Howard Hughes Medical Institute, UCLA, Los Angeles, CA 90095, USA; Department of Chemistry and Biochemistry, UCLA-DOE Institute, Molecular Biology Institute, UCLA, Los Angeles, CA 90095, USA; Department of Biological Chemistry, UCLA-DOE Institute, Molecular Biology Institute, UCLA, Los Angeles, CA 90095, USA; Howard Hughes Medical Institute, UCLA, Los Angeles, CA 90095, USA; Department of Chemistry and Biochemistry, UCLA-DOE Institute, Molecular Biology Institute, UCLA, Los Angeles, CA 90095, USA; Department of Biological Chemistry, UCLA-DOE Institute, Molecular Biology Institute, UCLA, Los Angeles, CA 90095, USA; Howard Hughes Medical Institute, UCLA, Los Angeles, CA 90095, USA; Department of Chemistry and Biochemistry, UCLA-DOE Institute, Molecular Biology Institute, UCLA, Los Angeles, CA 90095, USA; Department of Biological Chemistry, UCLA-DOE Institute, Molecular Biology Institute, UCLA, Los Angeles, CA 90095, USA; Howard Hughes Medical Institute, UCLA, Los Angeles, CA 90095, USA; Department of Chemistry and Biochemistry, UCLA-DOE Institute, Molecular Biology Institute, UCLA, Los Angeles, CA 90095, USA; Department of Biological Chemistry, UCLA-DOE Institute, Molecular Biology Institute, UCLA, Los Angeles, CA 90095, USA; Howard Hughes Medical Institute, UCLA, Los Angeles, CA 90095, USA; Department of Chemistry and Biochemistry, UCLA-DOE Institute, Molecular Biology Institute, UCLA, Los Angeles, CA 90095, USA; Department of Biological Chemistry, UCLA-DOE Institute, Molecular Biology Institute, UCLA, Los Angeles, CA 90095, USA; Howard Hughes Medical Institute, UCLA, Los Angeles, CA 90095, USA

**Keywords:** amyloid, cryo-electron microscopy, mutation, Charcot–Marie–Tooth disease, hereditary motor and sensory neuropathy with proximal dominant involvement

## Abstract

We previously presented a bioinformatic method for identifying diseases that arise from a mutation in a protein's low-complexity domain that drives the protein into pathogenic amyloid fibrils. One protein so identified was the tropomyosin-receptor kinase–fused gene protein (TRK-fused gene protein or TFG). Mutations in TFG are associated with degenerative neurological conditions. Here, we present experimental evidence that confirms our prediction that these conditions are amyloid-related. We find that the low-complexity domain of TFG containing the disease-related mutations G269V or P285L forms amyloid fibrils, and we determine their structures using cryo-electron microscopy (cryo-EM). These structures are unmistakably amyloid in nature and confirm the propensity of the mutant TFG low-complexity domain to form amyloid fibrils. Also, despite resulting from a pathogenic mutation, the fibril structures bear some similarities to other amyloid structures that are thought to be nonpathogenic and even functional, but there are other factors that support these structures' relevance to disease, including an increased propensity to form amyloid compared with the wild-type sequence, structure-stabilizing influence from the mutant residues themselves, and double-protofilament amyloid cores. Our findings elucidate two potentially disease-relevant structures of a previously unknown amyloid and also show how the structural features of pathogenic amyloid fibrils may not conform to the features commonly associated with pathogenicity.

**Significance Statement**

The Identification of Mutations Promoting Amyloidogenic Transitions (IMPAcT) method is a computational technique for identifying disease-related mutant proteins that form amyloid fibrils associated with pathology. In previous work, we showed that the IMPAcT method identifies disease-related mutant proteins previously known to form amyloid fibrils. It also predicts disease-related proteins to form amyloid fibrils, not previously known to do so. One of these is tropomyosin-receptor kinase–fused gene protein (TFG), whose mutant forms are associated with degenerative nerve diseases. Here, we confirm that mutant TFG forms amyloid fibrils and determine the molecular structures of the amyloid fibrils by cryo-electron microscopy. Our results validate the IMPAcT method and uncover how the TFG mutations affect the structure of the protein.

## Introduction

Previously, we developed a method to search for pathogenic mutations that induce amyloid aggregation of an otherwise functional protein (1). We have since named this method the Identification of Mutations Promoting Amyloidogenic Transitions method, or the IMPAcT method. Briefly, the method entails comparing the computed amyloidogenic propensity of mutant protein sequences to their wild-type (WT) counterparts and retaining those mutations that generate an amyloidogenic sequence from a nonamyloidogenic WT sequence. With the assumption that low-complexity domains (LCDs) would be prime candidates for amyloidogenic gain of function due to commonly functioning in-protein self-association and often being intrinsically disordered, we applied this method to every LCD in the human proteome and identified many candidate mutations in proteins not previously shown to be amyloidogenic. We demonstrated that two of these mutations, both in the tropomyosin-receptor kinase (TRK)-fused gene protein (TFG), do indeed induce fibrillar aggregates with amyloid characteristics.

**Fig. 1.**
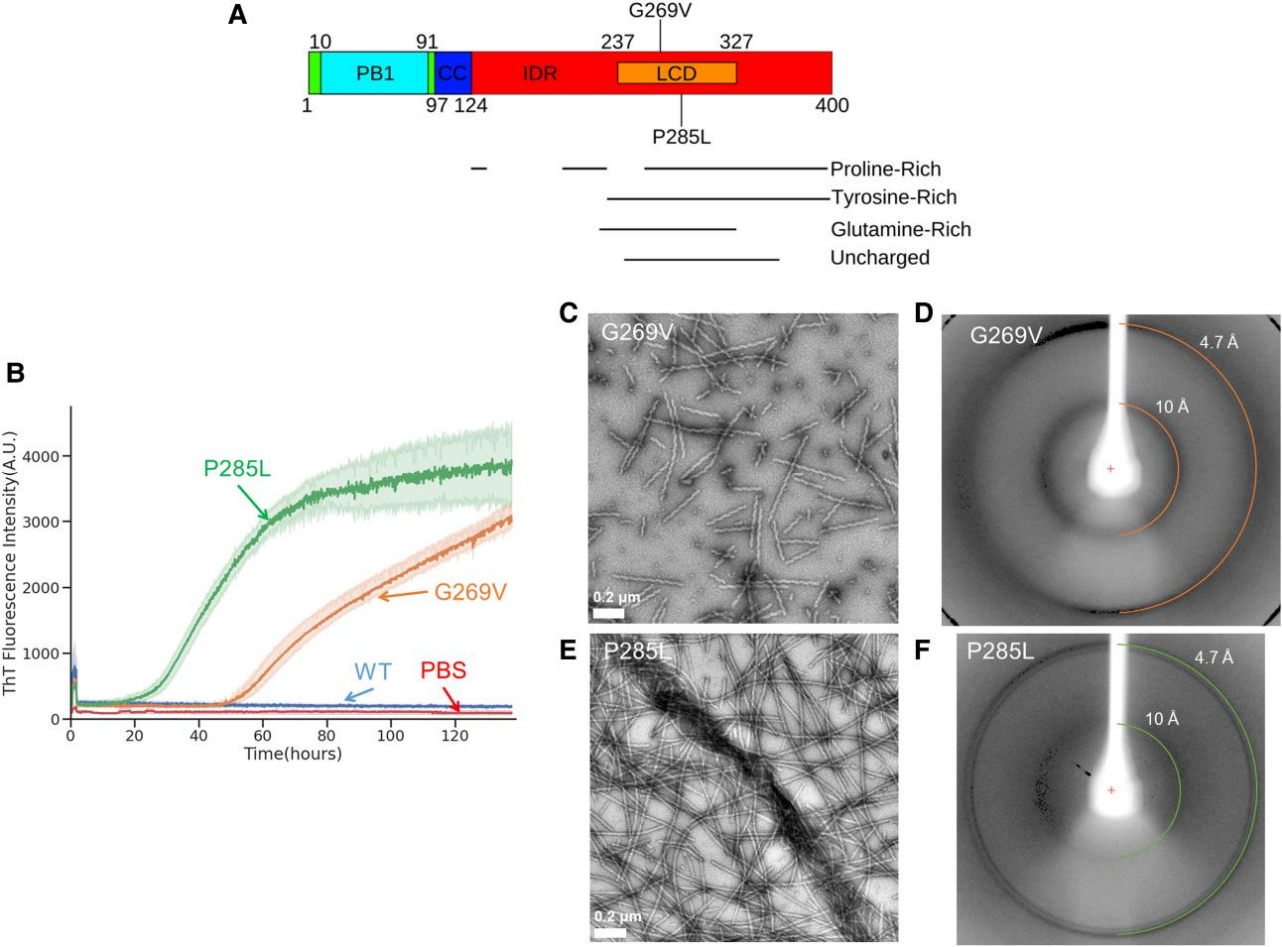
The TFG LCD forms amyloid fibrils. A) Domain structure of the full-length TFG protein. The protein contains an ordered PB1 domain and coiled-coil motif (residues 10–91 and 97–124, respectively), and the rest of the protein is intrinsically disordered. An LCD was identified using SEG (4) (residues 237–327), which was the portion of the protein that was conjugated to mCherry and expressed and purified. Amyloid-promoting mutations G269V and P285L are also labeled. The ordered fibril cores of both mutant fibrils encompass both of these mutation locations. Regions of the protein especially enriched for certain amino acids are also indicated. B) Time-dependent ThT fluorescence for the WT TFG LCD sequence conjugated to a molecule of mCherry (mC-TFG-LCD, labeled “WT”), the TFG LCD with the G269V mutation conjugated to a molecule of mCherry (mC-TFG-LCD-G269V, labeled “G269V”), the TFG LCD with the P285L mutation conjugated to a molecule of mCherry (mC-TFG-LCD-P285L, labeled “P285L”), and PBS buffer (labeled “PBS”). All constructs are at 50 μM concentration in PBS with ThT at 40 μM concentration. The mC-TFG-LCD-G269V construct and the PBS blank have *n* = 3 technical replicates and the mC-TFG-LCD and mC-TFG-LCD-P285L constructs have *n* = 6 technical replicates. The *y*-axis values represent the mean ThT fluorescence value of all replicates for each construct. C and E) Electron micrograph of the mC-TFG-LCD-G269V sample (C) and the mC-TFG-LCD-P285L sample (E) at the endpoint of the ThT assay. The mC-TFG-LCD construct did not form fibrils. D and F) X-ray fiber diffraction of mC-TFG-LCD-G269V fibrils (D) and mC-TFG-LCD-P285L fibrils (F). Rings are present at 4.7 and 10 Å spacing with distinct wedges, indicative of a cross-β structure.

TFG functions in the early secretory pathway by regulating the transport of secretory vesicles between the endoplasmic reticulum (ER) and ER–Golgi intermediate compartments (ERGICs) (2). More specifically, TFG brings together secretory vesicles from the ER by interacting with the conserved coat protein complex II (COPII) on the surface of the vesicles (3). This interaction persists until the vesicles tether and fuse with the ERGIC membrane. Thus, the TFG–COPII interaction is crucial for preventing the diffusion of COPII-coated vesicles away from the ER–ERGIC interface, an event that would derail protein secretion. To perform its role in clustering vesicles, TFG self-assembles into homomeric cup-shaped octamers via interactions encoded in its N-terminal Phox and Bem1p (PB1) domain and a subsequent coiled-coil motif. However, the majority of TFG's 400 amino acids compose an intrinsically disordered region. Within this region is an LCD (residues 237–327) rich in proline, tyrosine, and glutamine (Fig. 1A) that functions to further polymerize these octamers into larger assemblies and phase-separate (2). Thus, TFG functions at very high local concentrations because of its self-interactions.

Mutations in the TFG LCD are associated with degenerative neurological disorders such as Charcot–Marie–Tooth disease type 2 (CMT2) and hereditary motor and sensory neuropathy with proximal dominant involvement (HMSN-P) (5, 6). CMT2 is a group of axonal neuropathies, typically with autosomal-dominant inheritance, characterized by progressive axonal degeneration and deterioration of muscle strength leading to distal wasting, weakness, sensory loss with reduced tendon reflexes, and foot deformity (7). HMSN-P is also a slow, progressive axonal degeneration disease with autosomal-dominant inheritance and is characterized by painful muscle cramps and muscle weakness in early stages, sensory disturbances (dysesthesia, hypesthesia, and hypopallesthesia), and, in late stages, virtually no muscle strength in the extremities, making patients unable to walk and bedridden with impaired respiration similar to amyotrophic lateral sclerosis (ALS) (8). The mutations associated with these diseases have also been shown to lead to an aggregation of TFG in cells (5, 6).

In this study, we determined the cryo-electron microscopy (cryo-EM) structure of the amyloid fibers produced by the TFG LCD with both of the disease mutants identified by the IMPAcT method, G269V and P285L, which are associated with CMT2 (5) and HMSN-P (6), respectively. These structures further validate the amyloid propensity of this protein, elucidate a potential molecular basis for the effects of these mutations on disease, and add nuance to the paradigm of structural features that evince either pathogenic or nonpathogenic amyloids.

## Results

### Fibril formation of the TFG LCD

We expressed and purified the LCD of TFG (residues 237–327) conjugated to a molecule of mCherry, for enhanced solubility, with the WT TFG sequence (mC-TFG-LCD), containing the G269V mutation (mC-TFG-LCD-G269V), or containing the P285L mutation (mC-TFG-LCD-P285L). We shook the constructs at a concentration of 50 μM at 37°C for 138 h in the presence of the amyloidophilic dye Thioflavin T (ThT) at a concentration of 40 μM to monitor amyloid fibril formation in vitro. mC-TFG-LCD-G269V and mC-TFG-LCD-P285L formed ThT-positive aggregates, while mC-TFG-LCD did not (Fig. 1B). The mutant protein fibrils were visualized with negative stain electron microscopy (EM) (Fig. 1C and E) and the cross-β nature of the fibrils was confirmed via X-ray diffraction of aligned and dried fibrils (Fig. 1D and F).

### Cryo-EM structure of mC-TFG-LCD mutant fibrils

**Fig. 2.**
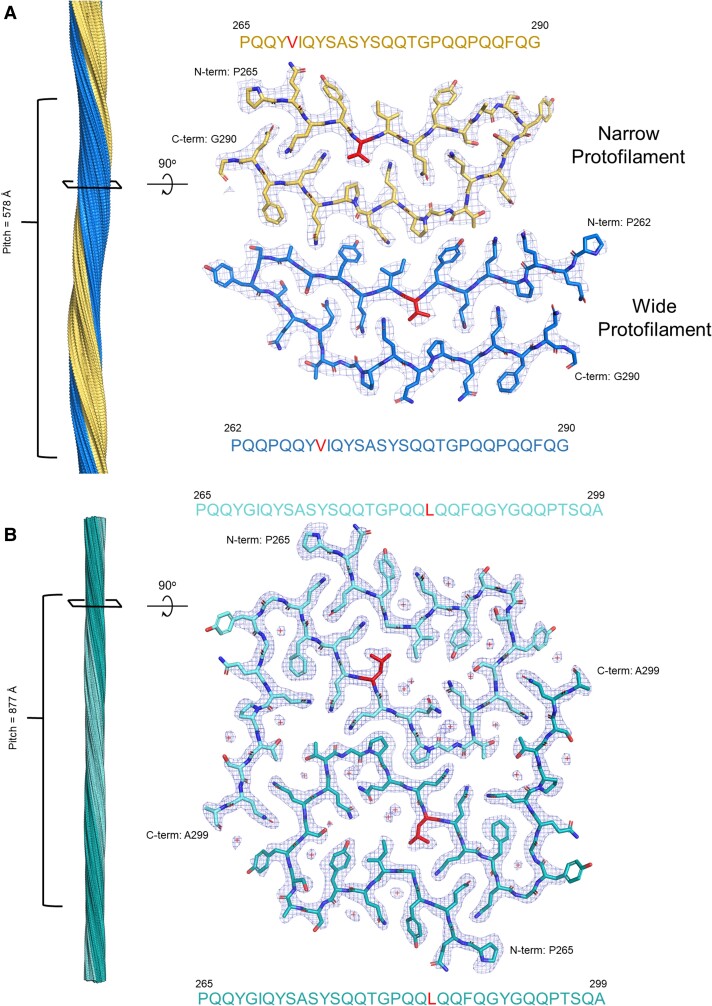
Cryo-EM structures of the mC-TFG-LCD-G269V and mC-TFG-LCD-P285L amyloid fibril cores. A) Left: mC-TFG-LCD-G269V fibril reconstruction showing a left-handed twist and the pitch. Right: Density map and atomic model of one layer of the fibril viewed down the fibril axis. The G269V mutation is red. The sequence context is _265_PQQY***V***IQYS_273_. The protofilament with three extra residues resolved (P262–Q264) is referred to as the “wide” protofilament and the other protofilament, starting at residue 265, is accordingly referred to as the “narrow” protofilament. B) Left: mC-TFG-LCD-P285L fibril reconstruction showing a left-handed twist and the pitch. Right: Density map and atomic model of one layer of the fibril viewed down the fibril axis. The P285L mutation is red. The sequence context is _281_GPQQ***L***QQFQ_289_. The plus symbols in the density represent modeled water molecules.

To understand the relationship of the G269V and P285L disease mutations with the formation of fibrils and further confirm their amyloid nature, we determined the cryo-EM structure of the mC-TFG-LCD-G269V fibrils to a resolution of 2.8 Å (Fig. 2A) and the mC-TFG-LCD-P285L fibrils to a resolution of 2.6 Å (Fig. 2B). Data collection and refinement statistics for mC-TFG-LCD-G269V and mC-TFG-LCD-P285L are summarized in Table 1.

**Table 1.** Cryo-EM data collection, refinement, and validation statistics.

	G269V mutant (EMDB-41195) (PDB 8TEQ)	P285L mutant (EMDB-41198) (PDB 8TER)
*Data collection and processing*		
Magnification	×105,000	×81,000
Voltage (kV)	300	300
Electron exposure (*e*^−^/Å^2^)	50	50
Defocus range (μm)	−1.8 to −2.6	−1.8 to −2.6
Pixel size (Å)	0.86 (counting mode)	0.539 (super mode)
Symmetry imposed	C1; Helical	C1; Helical
Initial particle images (no.)	384,324	690,373
Final particle images (no.)	31,643	193,640
Map resolution (Å)	2.8	2.6
FSC threshold	0.143	0.143
Map resolution range (Å)	200–2.8	200–2.6
Helical twist (°)	−3.07	179.02
Helical rise (Å)	4.93	2.39
*Refinement*		
Initial model used (PDB code)	De novo	De novo
Model resolution (Å)	2.8	2.6
FSC threshold	0.5	0.5
Model resolution range (Å)	200–2.8	200–2.6
Map sharpening *B* factor (Å^2^)	103.86	143.57
*Model composition*		
Nonhydrogen atoms	6,705	5,780
Protein residues	825	700
*B factors (Å^2^)*		
Protein	44.55	14.16
*RMS deviations*		
Bond lengths (Å)	0.003	0.007
Bond angles (°)	0.561	0.894
*Validation*		
MolProbity score	1.37	1.95
Clashscore	6.75	10.67
Poor rotamers (%)	0.00	0.00
*Ramachandran plot*		
Favored (%)	98.04	93.94
Allowed (%)	1.96	6.06
Disallowed (%)	0.00	0.00

The mC-TFG-LCD-G269V fibrils have a uniform morphology consisting of two protofilaments of the same sequence (although one has three more residues resolved than the other) but slightly different structures and interacting through a class 4 zipper interface (9, 10) (parallel, face-to-back, up-down packing; Fig. 2A). The fibril has a pitch of 578 Å, a left-handed helical twist of −3.07°, and a helical rise of 4.93 Å. As expected from the initial biochemical evidence, these features are all consistent with identifying this structure as an amyloid fibril. The fibril core contains 2 protofilaments, 1 with a wide core of 29 residues (P262–G290) and 1 with a narrow core of 26 residues (P265–G290; 3 fewer residues resolved; Fig. 2A). Each protofilament consists of a single dagger-shaped β-arch and constitutes <30% of the LCD (Fig. 1A).

The disease-relevant mutation G269V is present and buried in the interiors of both protofilaments. V269 resides at the center of the longest β-strand of each protofilament (Fig. 2A) and fits tightly with side chains from the opposite arm of the β-arch. Thus, V269 excludes water from the interior of the protofilaments and contributes to a heterotypic steric zipper. Moreover, as a participant in the only hydrophobic interaction in the interior of each protofilament, V269 contributes strongly to the energetic stability of the β-arch and at the protofilament interface.

The mC-TFG-LCD-P285L fibrils have two distinct polymorphs: a narrow, untwisting fibril (making up the minority of fibrils) whose structure could not be determined, and the major polymorph, which consists of two identical protofilaments related by a pseudo 2_1_ screw axis, forming a class 1 zipper interface (parallel, face-to-face, up-up packing; Fig. 2B). The fibril has a pitch of 877 Å, a left-handed helical twist of 179.02°, and a helical rise of 2.39 Å. As expected, these features align with the biochemical evidence of amyloid fibrils. Both protofilaments consist of 35 residues (P265–A299) and form shapes reminiscent of the lower-case letters “d” and “p.”

The disease-relevant mutation P285L is present and buried in the protofilament interior. L285 is a member of the longest β-strand of the protofilament core, fills in the space left by the glycine residue across from it, and forms a hydrophobic interaction with I270. Water molecules resolved in the interior of the fiber core reveal that L285 forms a barrier between solvent-accessible surface area and a glutamine zipper. Q283 is interacting with three water molecules, while the zipper made up of Q267, Q287, and Q289 is protected by L285 from solvent infiltration.

### Structural differences are directly attributable to the effects of mutant residues

The fibril cores formed by mC-TFG-LCD-G269V and mC-TFG-LCD-P285L have distinctly different folds. The most consistent feature between the two is the glutamine steric zipper formed by residues Q267, Q287, and Q289 (Fig. S1). However, only the narrow protofilament of the mC-TFG-LCD-G269V fibril exhibits this structural motif, while the wide protofilament has a different arrangement of these glutamine residues in relation to each other. Besides this similarity, the portion of the core from G/V269 to P/L285 has a distinct shape in each structure and also the mC-TFG-LCD-P285L fibril core has several extra C-terminal residues resolved (Y291–A299) and these residues form both intra- and interprotofilament interactions.

The difference in the shape of the 269–285 segment is attributed to the different residues terminating this section in either structure. In the mC-TFG-LCD-G269V structure, there is a valine at position 269 that participates in a β-strand from Q266 to S273, but in the mC-TFG-LCD-P285L structure, there is a glycine at position 269, which allows more flexibility in the peptide backbone, creating a slight kink that disrupts the β-strand and flips I270 from solvent-facing to the protofilament interior, allowing it to interact with L285, the mutant residue. This kink changes the shape of this segment from a dagger-like fold to a wider, flattened out turn that has a solvent-accessible channel. The G269V mutation contributes to a longer β-strand that constrains the core to a more dagger-shaped fold, while the P285L mutation stabilizes the kink centered on G269 by introducing a hydrophobic interaction with I270.

The shape difference of the 269–285 segment also contributes to the presence or absence of Y291–A299 in the resolved fibril core. This is due to the fact that, in the mC-TFG-LCD-P285L structure, residues T296–Q298 form a short polar zipper with residues Q278–T280 on the opposite protofilament. Q278–T280 are constituents of a short β-strand which, in the mC-TFG-LCD-P285L structure, is almost orthogonal to the central protofilament interface, while in the mC-TFG-LCD-G269V structure, it is almost parallel to the protofilament interface. This makes the 296–298 segment available to interact with residues 296–298 only in the P285L structure because of the shape of the entire 269–285 segment, and this shape is attributable to the type of mutation that the protein has.

**Fig. 3.**
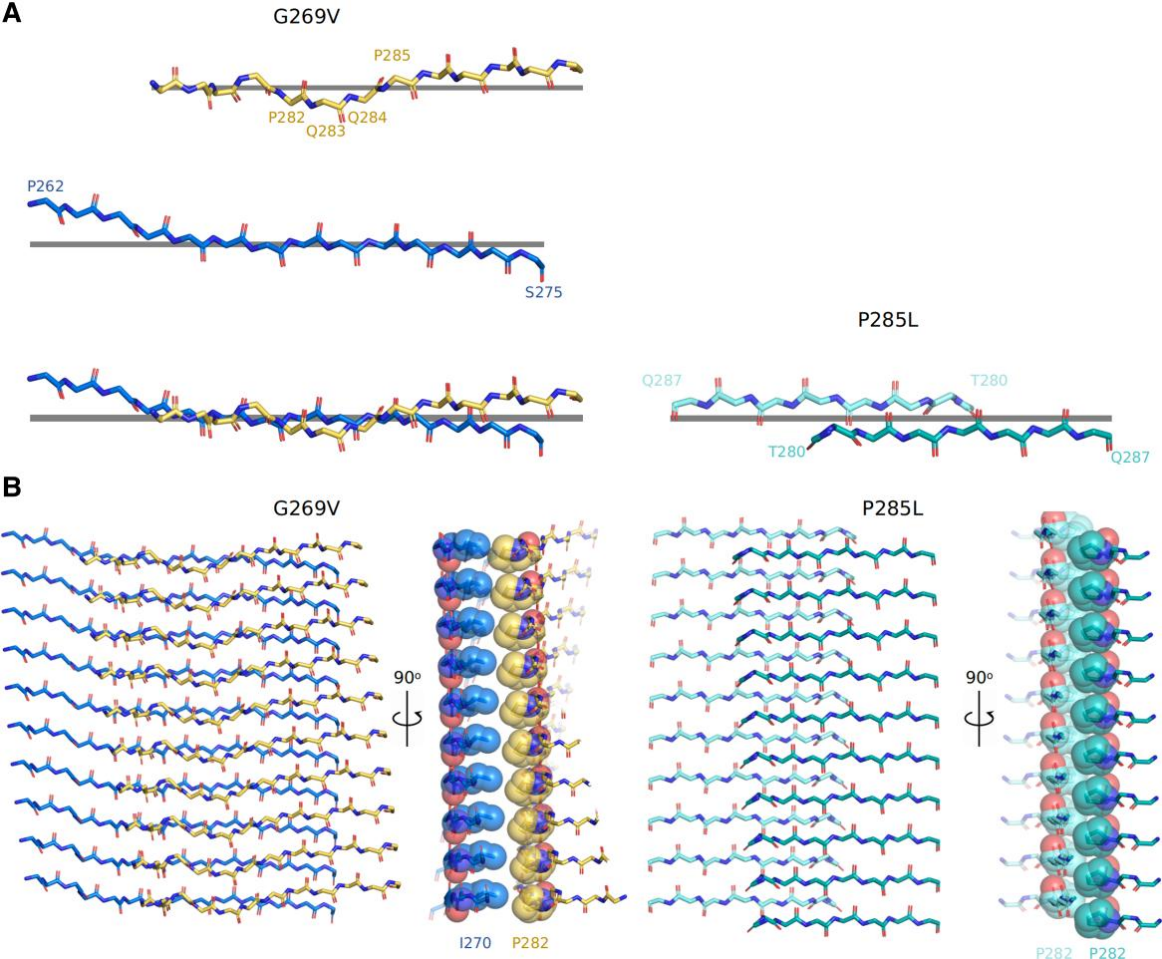
Protofilament interfaces of mC-TFG-LCD-G269V and mC-TFG-LCD-P285L fibrils. A) Top: The peptide backbone of the narrow protofilament of the mC-TFG-LCD-G269V fibril that constitutes the protofilament interface (residues 278–290) viewed orthogonal to the fibril axis with a line representing the plane perpendicular to the fibril axis. Residues P282–P285 are labeled to highlight the backbone warp that they constitute. Middle: The peptide backbone of the wide protofilament of the mC-TFG-LCD-G269V fibril that constitutes the protofilament interface (residues 262–275) viewed orthogonal to the fibril axis with a line representing the plane perpendicular to the fibril axis. Bottom left: The peptide backbones of the aforementioned segments of the narrow and wide protofilaments overlaid relative to each other as they exist in the mC-TFG-LCD-G269V fibril structure with a line representing the plane perpendicular to the fibril axis. The peptide backbones of each protofilament are only in plane with each other at a few spots, and notably not at the center of the interface due to the backbone warp of the narrow protofilament at residues 282–285. Bottom right: The peptide backbones of T280–Q287 of both protofilaments of the mC-TFG-LCD-P285L fibril overlaid relative to each other as they exist in the mC-TFG-LCD-P285L fibril structure with a line representing the plane perpendicular to the fibril axis. These protofilaments are offset from each other due to the pseudo 2_1_ screw axis of symmetry of the protofilaments. B) Left: multiple layers of the mC-TFG-LCD-G269V fibril, showing how the protofilaments are not offset from each other in terms of overall symmetry despite the backbone warp. Van der Waals spheres are shown for residues I270 of the wide protofilament and P282 of the narrow protofilament. Right: multiple layers of the mC-TFG-LCD-P285L fibril showing the offset of each layer due to the pseudo 2_1_ screw axis of symmetry. Van der Waals spheres are shown for residue P282 in both protofilaments.

The last major difference between the two structures is that the mC-TFG-LCD-G269V structure has asymmetric protofilaments not related by a screw axis, while the mC-TFG-LCD-P285L structure has symmetrical protofilaments related by a pseudo 2_1_ screw axis. Without a screw axis, the protofilament interface is in a single plane, but with a screw axis, the layers of either protofilament are vertically offset from each other. Screw axes are prevalent in amyloid fibril structures likely due to improved space-filling at the protofilament interface relative to interfaces not related by screw symmetry (11) (Fig. 3B). This symmetry difference is difficult to intuitively link to the mutations, but a difference in the structures of the protofilament interfaces can indeed be attributed to the difference in mutations. The narrow protofilament of the mC-TFG-LCD-G269V fibril has two proline residues in the segment that makes up the protofilament interface. Looking down the fibril axis, these proline residues seem to be in a relatively straight segment resembling a typical β-strand, which is unusual for a proline-rich segment. However, when viewed orthogonal to the fibril axis, this segment has some obvious buckling, causing the peptide backbone to have a wave-like shape, apparently a side-effect of having proline resides in a β-strand-like conformation in one dimension (Fig. 3A). This warp in the backbone shape, along with a slight opposite angling of the planes of each individual protofilament, causes the zipper interface to be somewhat staggered in the mC-TFG-LCD-G269V fibril despite not being related by a screw axis. The G269V mutation may stabilize this unusual configuration by forming a hydrophobic interaction with P285 and thus forcing the P282–P285 segment to conform to a β-sheet-like interaction in one dimension and warp in another dimension. The mC-TFG-LCD-P285L fibril does not exhibit this buckle in the peptide backbone, because the corresponding proline residues (P282 and P285) are either involved in a turn that does not exist in the mC-TFG-LCD-G269V fibril (P282 participates in a turn) or mutated to a different residue (P285 is mutated to leucine), and therefore, they do not cause the segment to buckle. Thus, both structures exhibit a staggered zipper interface, but the staggering is related to two different structural mechanisms that can be indirectly linked to the mutant residues. It is unclear, however, why the mC-TFG-LCD-G269V fibril does not also form with a pseudo 2_1_ screw axis.

### Energetic analysis reveals resemblance to reversible, functional amyloids

**Fig. 4.**
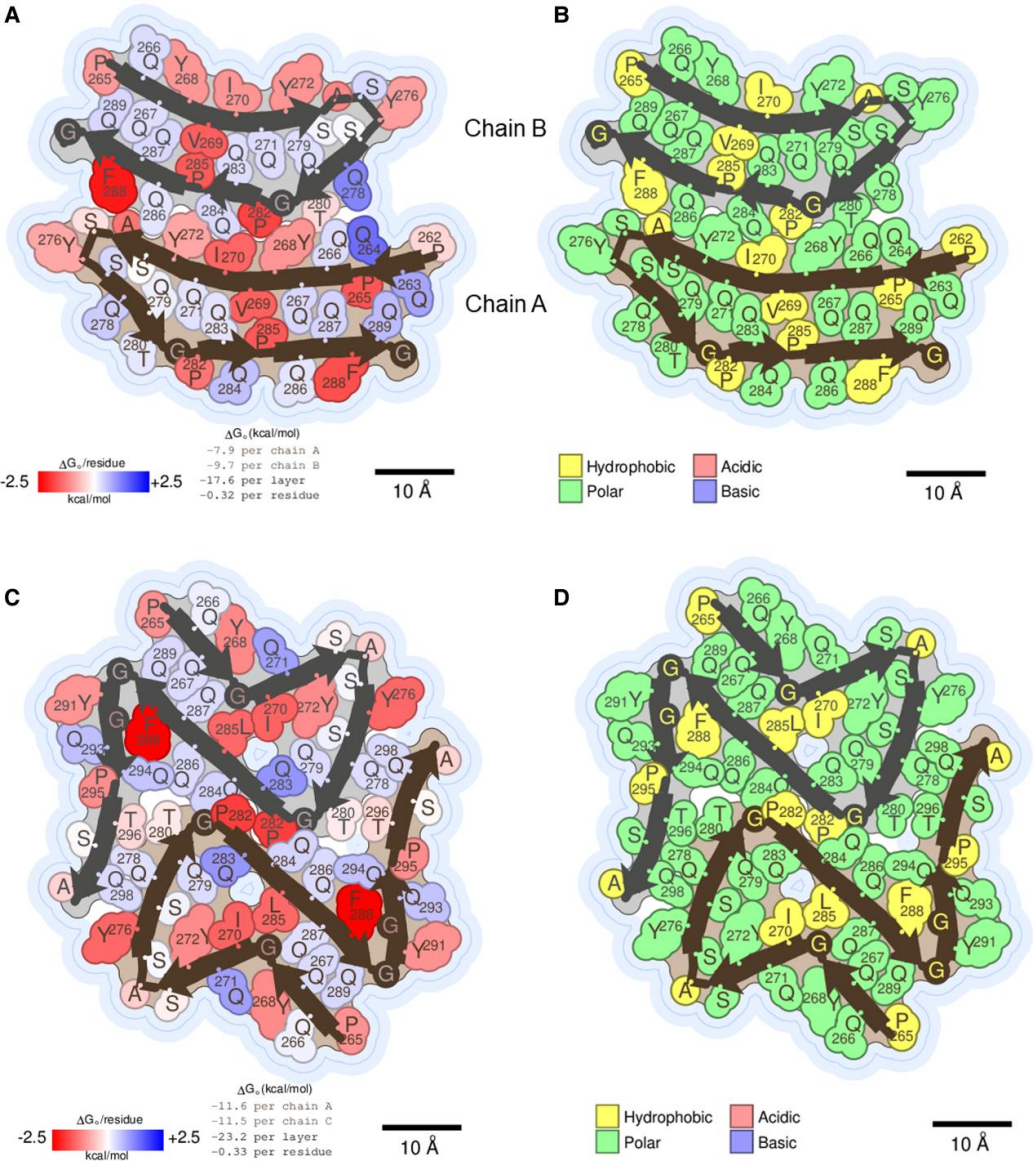
Solvation energy maps and polarity maps of the mutant TFG LCD amyloid fibrils. A and C) Solvation energy map of the mC-TFG-LCD-G269V amyloid fibril (A) and mC-TFG-LCD-P285L amyloid fibril (C). Residues are colored according to their stabilization energies. Deeper blue (positive) is unfavorable for amyloid assembly and deeper red (negative) is favorable. The thin dark blue line represents the solvent-accessible surface. Energy values are listed below the structure illustration. For the mC-TFG-LCD-G269V fibril, chain A is the wide protofilament and chain B is the narrow protofilament in the illustration. B and D) Polarity map of the mC-TFG-LCD-G269V amyloid fibril (B) and mC-TFG-LCD-P285L amyloid fibril (D). Residues are colored according to whether they are polar, hydrophobic, acidic, or basic. The thin dark blue line represents the solvent-accessible surface.

We calculated the atomic solvation energy of the mC-TFG-LCD-G269V and mC-TFG-LCD-P285L fibrils using the coordinates of the ordered fibril cores (Fig. 4A and C) and compared their values with other amyloid fibril structures. The solvation energy per residue is −0.32 kcal mol^−1^ for mC-TFG-LCD-G269V and −0.33 kcal mol^−1^ for mC-TFG-LCD-P285L, both of which are similar to the reversible hnRNPA2 WT LCD fibril (−0.34 kcal mol^−1^ per residue) (12). The solvation energy per layer is −17.6 kcal mol^−1^ for mC-TFG-LCD-G269V and −23.2 kcal mol^−1^ for mC-TFG-LCD-P285L, which are also similar to the hnRNPA2 WT LCD (−19.5 kcal mol^−1^) (12). Other amyloid structures with similar calculated energies belong to the fused in sarcoma (FUS)-LCD (Protein Data Bank [PDB] identifier (ID): 5w3n; energy per residue: −0.20 kcal mol^−1^; energy per layer: −12.2 kcal mol^−1^) (13), the fungal prion Het-s (PDB ID: 2rnm; energy per residue: −0.30 kcal mol^−1^; energy per layer: −18.8 kcal mol^−1^) (13), and a heparin-induced recombinant tau fiber “tau 4R snake” (PDB ID: 6qjh; energy per residue: −0.30 kcal mol^−1^; energy per layer: −17.7 kcal mol^−1^) (13). The FUS-LCD structure is ostensibly functional and reversible (14), but Het-s, while functional, is not reversible (15), and tau 4R snake does not seem to be functional, but it is not representative of the structures of tau fibers extracted from patient brains (16), and its reversibility is unknown. Definitively pathogenic structures tend to have significantly more stable solvation energy values, such as for transthyretin (TTR) fibers (PDB ID: 6sdz; energy per residue: −0.68 kcal mol^−1^; energy per layer: −62.1 kcal mol^−1^) (13) and serum amyloid A (PDB ID: 6mst; energy per residue: −0.65 kcal mol^−1^; energy per layer: −68.8 kcal mol^−1^) (13). Thus, based on solvation energy, we would group the mC-TFG-LCD-G269V and mC-TFG-LCD-P285L amyloid fibers with functional and/or reversible amyloids rather than pathogenic ones.

One explanation why our energetic algorithm predicts relatively poor stability for the fibril cores is the abundance of polar residues that form the inter- and intramolecular steric zippers in each structure, mainly glutamine residues (Fig. 4B and D). Pathogenic amyloids are better stabilized by hydrophobic interactions in their cores than by polar interactions (13). The dominance of glutamine in the fibril cores resembles the glutamine-rich β-arches of the reversible, functional drosophila amyloid Orb2 (17) (Fig. S2). However, glutamine zippers are also found in pathogenic amyloids such as in some transactive response (TAR) DNA-binding protein 43 (TDP-43) fibrils (18) and aggregates of huntingtin (19). In both mutant fibrils, the glutamine side chains in the interior of each protofilament are positioned such that they form hydrogen bonds with the glutamines above and below them along the fibril axis (Fig. S3). Also, in the mC-TFG-LCD-G269V fibril, Q278 of the narrow protofilament and Q264 of the wide protofilament form a hydrogen bond between the two protofilaments in addition to interlayer hydrogen bonds (Fig. S3A). In the mC-TFG-LCD-P285L fibril, an intra-protofilament hydrogen bond ladder is formed between Q286 and Q294 (Fig. S3B). Interestingly, in the mC-TFG-LCD-P285L structure, one of the interior glutamine residues does not participate in interlayer hydrogen bonding because it is being solvated by water molecules. Overall, this hydrogen bonding activity may compensate for the destabilizing effect of their hydrophilicity.

Another destabilizing feature of the mutant fibrils is the abundance of proline residues within the fibril cores. Prolines weaken β-sheet formation owing to their inability to donate a hydrogen bond to a backbone carbonyl oxygen in an adjacent layer of the fibril—a consequence of prolines’ unique side chain being covalently bonded to the peptide backbone amide. In the mC-TFG-LCD-G269V fibril, the wider protofilament core contains four prolines and the narrower one contains three; one of these prolines (P285 in both protofilaments) is directly across from the mutant valine residue, participating in a hydrophobic interaction; thus, the valine is compensating for the structural weakness incurred by the presence of the proline residue. In the mC-TFG-LCD-P285L fibril, both protofilaments contain three prolines and one of them (P282) participates in the protofilament interface and thus is able to somewhat contribute to the structural stability of the fibril. The TFG LCD as a whole is relatively proline-rich, containing 14 prolines among its 90 amino acids. The presence of these prolines within the fibril core would significantly destabilize this fibril because of its lack of hydrogen bonding capability (20) but may be compensated for by the hydrogen bonding of the glutamine side chains, as described above.

One feature that distinguishes the mutant TFG fibrils from reversible amyloids, however, is the presence of two protofilaments. The most reversible amyloid structures consist of only a single protofilament, which may make them more amenable to disassembly (13). The double-protofilament structure also bolsters stability by compensating for the meager energy per chain (∼−9.0 kcal mol^−1^ for mC-TFG-LCD-G269V and ∼−11.5 kcal mol^−1^ for mC-TFG-LCD-P285L), which is poor due to the previously mentioned energetic penalty of desolvating glutamine side chains as well as the relatively low number of amino acids in each protofilament. The bundling of two protofilaments is a feature that is more common to pathogenic amyloids, and every structure of an amyloid known to be conditionally labile has a single-protofilament core (13).

**Fig. 5.**
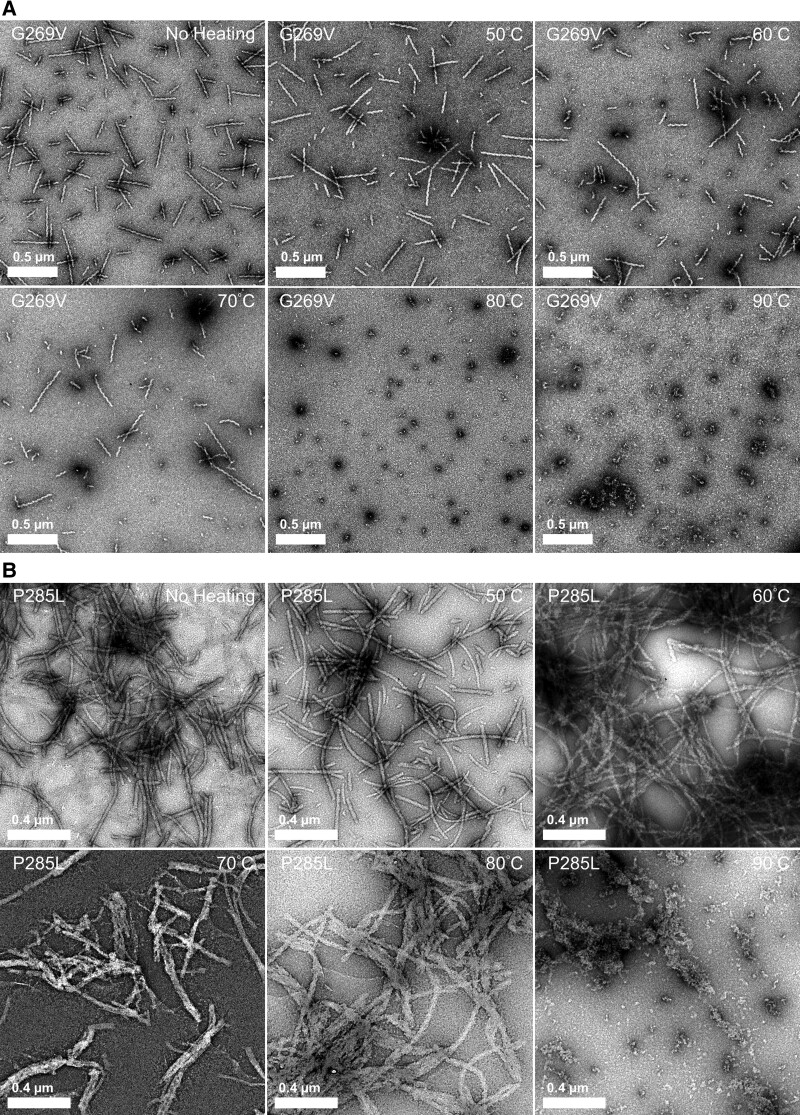
Heat stability of mutant TFG LCD amyloid fibers. Amyloid fibers formed at 50 μm monomer concentration at 37°C were diluted 1:5 in PBS and distributed into 10 μL aliquots. Each aliquot was heated to the specified temperature (40 to 100°C in intervals of 10°C) for 10 min each and then each sample was prepared for negative stain EM. A) Representative images of mC-TFG-LCD-G269V fibrils heated to 50 to 90°C. Fibrils remain clearly visible in all samples heated below 80°C but are completely absent in samples heated to 80°C and above. B) Representative images of mC-TFG-LCD-P285L fibrils heated to 50 to 90°C. Fibrils remain clearly visible in all samples heated below 90°C but are completely absent in samples heated to 90°C and above.

To empirically test the stability of the mutant TFG fibrils, we heated the aliquots of the fibrils at temperatures ranging from 40 to 100°C for 10 min each. For the mC-TFG-LCD-G269V fibrils, we found that the fibrils remained present after being heated to 70°C, but dissociated after being heated to 80°C (Fig. 5A). For the mC-TFG-LCD-P285L fibrils, fibrous aggregates were visible up to 80°C and disappeared only at 90°C, although even at 60°C, the fibrils appeared bristly (Fig. 5B). The same trend was observed in response to treatment with varying concentrations of guanidine hydrochloride (GdnHCl), a chemical denaturant (Fig. S4). For mC-TFG-LCD-G269V, the fibrils began dissociating but were still abundant in 2 M GdnHCl, the fibrils were extremely sparse in 2.5 M GdnHCl, and all fibrils had disappeared in 3 M GdnHCl (Fig. S4A and B). For mC-TFG-LCD-P285L, the fibrils remained abundant, but bristly, in 2 M GdnHCl, they appeared further dissociated but still abundant in 2.5 M GdnHCl, but they were extremely sparse in 3 M GdnHCl (Fig. S4C and D). These fibrils are also extremely long-lived ones in general, with samples stored at 4°C for over 2 years still containing fibrils (Fig. S5). These results suggest that both mutant fibrils are relatively stable and difficult to dissociate.

### Asymmetric C5 hydrogen bonding capability

One feature of the mC-TFG-LCD-G269V fibril that might favor reversibility of the amyloid fibril assembly is its capability to form C5 hydrogen bonds. C5 hydrogen bonds are intraresidue hydrogen bonds between the amide nitrogen (donor) and carbonyl oxygen (acceptor) of the same amino acid residue (21). In a typical pleated β-strand, the donor and acceptor are too far from each other to be able to share a hydrogen atom between them. But, this gap can close (as observed in about 13% of β-strands in the PDB [20]), enabling the internal hydrogen bond to form. The conformational adjustment required to close this gap is the flattening of the pleat of the β-strand (i.e. |*ϕ*| and |*ψ*| > 150°). The extended appearance of these flattened β-strands gave rise to the acronym EAGLS (extended amyloid-like glycine-rich low-complexity segments) to describe the presence of C5 hydrogen bonding regions in amyloid fibrils of low-complexity sequences such as HNRNPA1 and nup54 (22). C5 hydrogen bonds are hypothesized to contribute to the reversibility of amyloid fibrils since they compete with the intermolecular hydrogen bonds that hold amyloid fibrils together. Hence, the presence of C5 hydrogen bonds in an amyloid fibril might signal a reduced energetic penalty for amyloid dissociation (22).

We observe two examples of this extended (unpleated) β-strand conformation in mC-TFG-LCD-G269V fibrils that exhibit the geometry required for C5 hydrogen bonding and presumably contribute to lability. These residues are located in the wide protofilament at residues S273 and Q278 (Fig. S6). Because these residues occur in a low-complexity domain, they may be considered as a new example of EAGLS (22). Notably, the corresponding residues in the narrow protofilament are pleated, and not extended, suggesting that the narrow protofilament is more stable than the wide one. The different degrees of pleating/extension at S273 and Q278 contribute to the differences in the shape of the two protofilaments, with the narrow protofilament being slightly more curved. The difference in curvature is further supported by a relative swap in the positions of two glutamine side chains (Q267 and Q287). Our observation of EAGLS in the wide protofilament suggests that the narrow protofilament by itself might be nonpathogenic—easy to form and easy to disassemble. The combination of narrow and wide protofilaments revealed here suggests a stable assembly that is less prone to clearance from the cell.

### G269V and P285L mutations contribute to structural stability

Because these amyloid fibril structures result from particular pathogenic mutations (G269V and P285L) and the WT sequence did not form fibrils, it is relevant to ascertain how these amino acid changes contribute to the formation of these particular structures. In regard to the G269V mutation, valine preferentially forms a β-strand secondary structure and thus contributes to the stability of steric zippers (23). In the mC-TFG-LCD-G269V structure, V269 forms a hydrophobic interaction with the proline residue across from it (P285), and this is the only hydrophobic interaction in the interior of the individual protofilaments. In regard to the P285L mutation, leucine is not particularly prone to form a β-strand secondary structure, more likely participating in an α-helix secondary structure, but it is much more amenable to being in a β-strand than the WT proline residue. Leucine is also capable of interlayer hydrogen bonding of the peptide backbone, while proline is not. In the mC-TFG-LCD-P285L structure, L285 forms a hydrophobic interaction with the isoleucine residue across from it (I270), which helps stabilize the fold of the protofilament. Because hydrophobic interactions are more stabilizing for amyloid fibrils than polar interactions, the hydrophobic interactions formed by the mutant residues in both structures may be integral to the formation and persistence of these structures.

To understand why the WT sequence may not form these fibril structures, we modeled the WT sequence into the structure of the mC-TFG-LCD-G269V fibril and the mC-TFG-LCD-P285L fibril (Fig. S7A and B). When we do this with the mC-TFG-LCD-G269V fibril, we see that the potentially important hydrophobic interaction between residues 269 and 285 that the mutant valine residue participated in is abolished and a gap is open where water can fit and potentially destabilize the adjacent glutamine zippers (Fig. S7A). The missing piece of the steric zipper at the core of the two protofilaments is thus detrimental to the formation of the fibril structure that we observe for the G269V mutant. As for the mC-TFG-LCD-P285L structure, when the mutant leucine is replaced with the WT proline, P285 may still be able to form a hydrophobic interaction with I270, but not in such a way that blocks the solvent from accessing the adjacent glutamine zipper like how leucine is able to do (Fig. S7B). This gap may preclude the mC-TFG-LCD-P285L structure from forming from the WT sequence, similar to the mC-TFG-LCD-G269V structure. These considerations explain the relevance of the mutations to the observed structures and why they are not observed for the WT sequence.

## Discussion

### Validation of the IMPAcT method

The IMPAcT method identifies amyloidogenic mutations by comparing the amyloid propensity of WT and mutant protein sequences (1). Mutations that convert a nonamyloidogenic sequence to an amyloidogenic one are considered hits. The fibril structures presented in this work are formed by a protein with either of two of the mutations identified by the IMPAcT method as being amyloidogenic. The presence of the mutant residues in both the resolved fibril cores is an indication of the IMPAcT method's ability to predict these sequences' amyloid propensities, and more generally, the efficacy of the IMPAcT method to identify previously unrecognized amyloid-related conditions. As patient-derived tissues of TFG-related diseases become available, the discovery in them of fibrillar aggregates would add great support to the prediction of the IMPAcT method.

We also utilized the IMPAcT method to identify the amyloidogenicity of benign variants, variants of uncertain significance, and two other pathogenic variants in the LCD of TFG, all of which are available through UniProt (https://www.uniprot.org/uniprotkb/Q92734/variant-viewer). We found that out of 24 variants, only 7 were IMPAcT hits (Table S1 and Fig. S8). Three of these are pathogenic or likely pathogenic variants, namely, G269V and P285L, which were examined in this work, as well as Y276H. The other four are variants of uncertain significance, three of which are mutations of proline residues. All but one of the variants that were not hits are benign variants or variants of uncertain significance. The one pathogenic variant is G269D, which is a mutation in the same residue as the amyloidogenic G269V mutation, but the mutant residue in this case is charged, which is not amenable to amyloid fibril formation. These results further support the fact that the IMPAcT method does not generate spurious hits only because of the underlying protein sequence; this further demonstrates a correlation between pathogenic variants and predicted amyloidogenicity and also encourages a closer examination of some variants of uncertain significance in the TFG LCD for amyloidogenicity.

Noting this, the IMPAcT method identified many other mutations as amyloidogenic in proteins that have not previously been recognized as amyloidogenic. Some of the other interesting candidate proteins are prelamin-A/C (LMNA) (mutations: G602S and R624H) and CHCHD10 (mutation: P34S), which have been shown to aggregate when mutated (24, 25), and KRT74 (mutation: D482N), since other keratin proteins are amyloidogenic (26–29) and the KRT74 mutation is associated with a hypotrichosis of the scalp (30), which is a disorder that is sometimes associated with the amyloidosis of another protein called corneodesmosin (31).

The IMPAcT method can also be repeated with different parameters to obtain even more candidates. For example, in our usage, we applied the method to proteins with LCDs and to mutations only within LCDs, but this could be expanded to a different subset of the human proteome such as all proteins with intrinsically disordered regions or even potentially the entire human proteome. Also, the method employs the ZipperDB database that bases its scoring method on the energetic fit of a protein sequence to a particular steric zipper structure. The structural template that ZipperDB uses can be modified to different archetypal steric zipper classes or even to a custom steric zipper structure. Our validation of the initial implementation of the IMPAcT method is encouraging for the veracity of its other predictions and for the merit of implementing the method in the future with a broader scope or modified parameters.

### Functional or pathogenic amyloids

Because the fibril structures of TFG are derived from protein sequences containing disease-associated mutations, it may have been expected that the structures would resemble pathogenic amyloids. However, what we see instead are many similarities to reversible and/or functional amyloids. These similarities are comparable values of solvation energy, polar steric zippers, extended β-strands, and the presence of only a single polymorph (12). Despite these similarities, the structures do share similarities to pathogenic amyloids: multiple protofilaments and a near lack of kinked segments called low-complexity amyloid-like reversible kinked segments (LARKS) (32). On top of this, some of those features associated with reversible and/or functional amyloids are not unique to reversible and/or functional amyloids (poor solvation energy in irreversible Het-s fibers; polar steric zippers in TDP-43 fibers) and not all functional amyloids are reversible (Het-s RIPK1/3 33, Pmel17 34). Also, it is conceivable that a reversible amyloid fibril can be pathogenic. This distinction between functional and reversible amyloids is important, because features that suggest a fibril structure belongs to one of these groups do not compound to suggest it belongs to both groups. For example, the C5 hydrogen bonding capability of mC-TFG-LCD-G269V could be considered an evidence of reversibility, but this does not automatically make it an evidence of functionality as well.

Functional amyloids, reversible or not, have associated cellular mechanisms that have evolved to manage their aggregation and maintain their functions (35). Therefore, cellular machinery may not be as well-equipped to mount a response to amyloid fibrils made from a mutant protein that does not form them as part of its normal function, regardless of the potential reversibility of the fibril. Because TFG's normal function does not seem to require the formation of amyloid-like fibrils, the mutant fibrils' commonalities with functional or reversible amyloids may be misleading when judging their pathogenicity.

Another consideration is how much these mutant structures reflect potentially transient structures formed by the LCD during the normal functioning of TFG. The protein has been shown to be capable of phase separation (1) and its function has been proposed to require the formation of a membraneless compartment between the ER and the ERGIC in which COPII-coated vesicles move through (3). Phase separation behavior and the formation of membraneless organelles, especially when facilitated by intrinsically disordered regions of proteins, are thought to involve the otherwise disordered regions taking on a secondary structure that includes hydrogen bonding between β-strands (20). The formation of amyloids is mediated by the formation of expansive hydrogen bond networks between layered β-strands, and therefore, the ability of TFG to form amyloid fibers supports the proposition that the native function of its LCD involves the formation of β-strands connected by hydrogen bonds. Our observation that TFG forms amyloid fibrils is in line with numerous other examples of functional phase-separating proteins that also form amyloid fibrils (13, 14, 36, 37), which contribute to the same conclusion that a β-strand secondary structure and hydrogen bonding are important for liquid–liquid phase separation of some proteins.

### Potential mechanisms of pathogenicity

First, it should be noted that fibrillar TFG has not yet been demonstrated to be present in human beings. The structures determined in this work serve as a validation of the ability of the mutations identified by the IMPAcT method to exert an amyloidogenic effect on a previously unidentified amyloidogenic protein. These structures, however, are of unclear clinical relevance since they were formed entirely in vitro and fibrillar TFG has not yet been isolated from disease tissue. We anticipate that this validation of the IMPAcT method will lead to further research on other amyloid proteins identified by the method, which includes a closer examination of tissue from patients with the TFG mutations that were identified by the method and validated here. Further, while we cannot confirm the relevance of the structures determined here to human disease, we can discuss the potential pathogenic mechanisms of these fibrils if they are indeed formed by mutant TFG in humans.

The underlying reason for the association between amyloid fibrils and disease has not been fully understood. Amyloid fibrils themselves may be cytotoxic (38, 39), or oligomeric intermediates between monomers and fibers may be the pathogenic species (40, 41). Indeed, both could be true, with some diseases resulting from either aggregation state, depending on the protein or mutations involved. If fibrils of mutant TFG are able to dissociate, it is less likely that fiber-related cytotoxicity is the basis of the mutation's disease association. However, regardless of the reversibility of the fibrils, if the mutant protein preferentially forms an amyloid fibril rather than its functional structure, this would manifest indistinguishably from a more mundane loss-of-function mutation. TFG knockout mouse models develop neuromuscular junction degeneration (42), and thus, it is likely that even if the only effect of the TFG mutations is a preference for an inert or reversible amyloid fibril, it would be sufficient to explain the observed disease state. This is in addition to the possibility that the mutant TFG fibrils are indeed irreversible in cells and are cytotoxic. There are multiple ways by which the formation of these fibrils could cause disease, and elucidating the actual mechanism requires a further biochemical study of the protein, but loss of function due to aberrant amyloid formation seems sufficient to explain the mutation's role in disease.

### mCherry is unlikely to influence the fibril core structure

The addition of an mCherry tag to the LCD constructs was intended to ensure solubility and discourage amorphous aggregation, similar to previous work on similar constructs (12). This tag was not cleaved off at any point during experimentation, and thus, it is relevant to ask whether this tag affected the formation of the fibrils that we observed. It is unlikely that this tag significantly influenced the formation of fibers based on three lines of reasoning. First, the WT construct was not able to form any fibers, so it is unlikely that the mCherry is what drove fibril formation in the mutant construct. In fact, mCherry may have increased solubility and discouraged self-interaction relative to the full-length protein, since the full-length protein has a larger disordered region as well as a PB1 domain that functions in self-oligomerization of TFG. This evidence suggests that this construct would be selective for fiber structures with greater energetic favorability than the untagged full-length protein, since the full-length protein may be more prone to interact with itself and aggregate. Second, the end of the mCherry tag is 34 amino acids away from the residues that make up the fibril cores in our structures. These 34 amino acids include a short 6-residue linker and 28 residues of the TFG LCD that is intrinsically disordered (25 for the wide protofilament of mC-TFG-LCD-G269V, making that sequence 31 amino acids away from mCherry). This distance allows enough flexibility for the interaction of the residues included in the fibril cores to be uninfluenced by the mCherry tag. Third, the mCherry tag is completely unresolved in the final structures. This means that there is no single preferred interaction between the mCherry and the fibril cores.

### Summary

Here, we present two near-atomic resolution structures of the amyloid fibril core formed by the LCD of TFG with either of two pathogenic mutations. These structures are a further validation of the prediction by the IMPAcT method that these particular disease-related mutations are amyloid-promoting. The mutated residues were found to facilitate the stability of the fibril core structures and thus fibril formation may be an explanation for their relevance in disease. The fibrils also have similarities to functional and reversible amyloid fibrils, implying that these features are not sufficient to explain the difference between pathogenic amyloids and functional/reversible amyloids.

## Materials and methods

### Low-complexity region prediction

The amino acid sequence of TFG was evaluated for low complexity using the segmentation algorithm SEG (4) with default settings: window length = 12, trigger complexity 2.2, extension complexity 2.5. A sequence was determined to be a low-complexity domain if it contained at least 35 residues scored as low complexity, with at most 5 interrupting non-low-complexity residues.

### Protein expression and purification

Recombinant TFG (237–327) for the WT, G269V, and P285L forms was purified using a pHis-parallel-mCherry vector, using a previously described method (37). Briefly, protein was overexpressed in BL21(DE3) gold *Escherichia coli* cells. Cultures were grown to an OD_600_ = 0.4–0.8 and then induced with 0.5 M isopropyl β-D-1 thiogalactopyranoside (IPTG) overnight. The cells were pelleted by centrifugation and the clarified lysate was purified by nickel-nitrilotriacetic acid (Ni-NTA) columns followed by size exclusion chromatography and dialyzed into phosphate-buffered saline (PBS).

### In vitro aggregation assay

WT and mutant TFG LCD were diluted to 50 μM in 1X PBS containing ThT at 40 μM to a final volume of 150 μL in black Nunc 96-well optical bottom plates (Thermo Scientific). A single polytetrafluoroethylene (PTFE) bead (0.125-inch diameter) was added to each well to facilitate agitation. The plates were incubated in a microplate reader (FLUOstar OMEGA; BMG Labtech) for ∼138 h at 37°C with 700 rpm double orbital shaking. Fluorescent measurements were recorded every 15 min using *λ*_ex_ = 440 nm and λ_em_ = 480 nm. This was performed with *n* = 3 technical replicates of the mutant and *n* = 6 technical replicates of the WT.

### Transmission EM

A quantity of 10 μL of aggregated WT and mutant TFG samples (taken from in vitro aggregation experiments) were spotted onto a carbon film on 150 mesh copper grids (Electron Microscopy Sciences) and incubated for 4 min. The grids were stained with 10 μL uranyl acetate solution (2% w/v in water) for 4 min. Excess solution was removed by blotting and air-dried for 4 min. Transmission EM (TEM) images were acquired by using a JOEL 100CX TEM electron microscope at 100 kV.

### X-ray fiber diffraction

Aggregated samples of TFG were centrifuged at 15,000 rpm for 30 min and buffer was exchanged with water twice. The samples were suspended between two siliconized glass capillaries ∼1 mm apart, forming a bridge between the two capillaries. The sample was allowed to dry and the capillary was used to mount the aggregate in an X-ray beam from a Rigaku FR-E rotating anode X-ray generator for 8 min. The diffraction pattern was collected on an R-axis IV++ imaging plate detector.

### Heat sensitivity assay

TFG fibrils were formed at 37°C as described above. At the end point of shaking, the sample was diluted 1:5 in PBS and divided into 7 aliquots of 10 μL each. Each aliquot was individually heated to either 40, 50, 60, 70, 80, 90, or 100°C for 10 min using a BioRad T100 Thermocycler. The entire aliquot was then used to prepare a TEM grid as described above.

### GdnHCl sensitivity assay

TFG fibrils were formed as described above. The fibrils were diluted 1:5 in solutions of GdnHCl dissolved in PBS such that solutions of 0, 2, 2.5, 3, 3.5, 4, and 4.5 M GdnHCl were made containing equal concentrations of TFG fibrils for a final volume of 187.5 μL for each GdnHCl concentration. These solutions were transferred in triplicate in 50 μL aliquots to a black Nunc 384-well optical bottom plate (Thermo Scientific) to continue shaking under identical conditions to the fibril formation step, except without any PTFE beads, for 2.25 h. TEM grids were prepared from these samples as described above.

### Cryo-EM data collection, reconstruction, and model building

Two and a half microliters of mC-TFG-LCD-G269V or mC-TFG-LCD-P285L fibril solution were applied to a Quantifoil 1.2/1.3 electron microscope grid that was glow-discharged for 4 min. The grids were blotted with filter paper to remove excess sample and plunge-frozen into liquid ethane using a Vitrobot Mark IV (FEI).

For mC-TFG-LCD-G269V, the cryo-EM dataset was collected on a Titan Krios (Thermo Fisher Scientific) microscope equipped with a Bioquantum K3 (Gatan) camera located at the Stanford-SLAC Cryo-EM Center (S^2^C^2^). The microscope was operated at 300 kV acceleration voltage and a slit width of 20 eV. Movies were acquired with a nominal pixel size of 0.86 Å (×105,000 nominal magnification) with a dose per frame of ∼1.3 *e*^−^/Å^2^. A total of 40 frames were recorded for each movie (total dose per movie was 50 *e*^−^/Å^2^). Automated data collection was driven by E Pluribus Unum (EPU) automation software package (Thermo Fisher Scientific). Defocus was set to −1.8 to −2.6 μm. A total of 15,192 movies were collected.

For mC-TFG-LCD-P285L, the cryo-EM dataset was collected on a Titan Krios (Thermo Fisher Scientific) microscope equipped with a Bioquantum K3 (Gatan) camera. The microscope was operated at 300 kV acceleration voltage and a slit width of 20 eV. Cryo-EM movies were collected at a nominal magnification of × 81,000 with the camera operated at its super-resolution mode, yielding a pixel size of 0.539 Å. Automated data collection was done with SerialEM (43). Defocus was targeted to −1.8 to −2.6  μm. A total of 7,920 movies were collected.

For both datasets, motion correction and dose weighting was performed using Unblur (44) and contrast transfer function estimation was performed using CTFFIND 4.1.8 (45). For both datasets, a subset of fibril particles was picked manually using EMAN2 e2helixboxer.py (46), which was used to train crYOLO (47). The last automatically picked the rest of the particles. We used REgularized LIkelihood OptimizatioN (RELION) (48, 49) to perform particle extraction, 2D classification, helical reconstruction, and 3D refinement. Helical reconstruction was performed with a cylindrical reference (48). The 3D classification was performed using three classes, and by manually controlling the tau_fudge factor and healpix_order, the particles were separated into good and bad classes. To obtain a higher-resolution reconstruction, the best particles from 1,024-pixel box 3D classification were selected, and helical tubes corresponding to good particles were extracted using a box size of 432 pixels for mC-TFG-LCD-G269V fibrils or 384 pixels for mC-TFG-LCD-P285L fibrils. After several more rounds of 3D classification with a refinement of helical twist and rise, we then used the final subset of particles to perform high-resolution gold-standard refinement. The final overall resolution was estimated to be 2.8 Å for mC-TFG-LCD-G269V fibrils and 2.6 Å for mC-TFG-LCD-P285L fibrils based on the 0.143 Fourier shell correlation (FSC) resolution cutoff. We then sharpened the maps using phenix.auto_sharpen (50).

We built de novo atomic models into the sharpened maps using Coot (51). We refined the structures using phenix.real_space_refine (52) and validated them using phenix.comprehensive_validation (53, 54).

### Solvation energy calculation

The solvation energy was calculated based on an algorithm described previously (13, 55). The solvation energy for each residue was calculated by the sum of the products of the area buried for each atom and the corresponding atomic solvation parameters. The overall energy was calculated by the sum of energies of all residues. The colors assigned to each residue in the solvation energy map represent the sum of energies over all atoms in the residue.

## Supplementary Material

Supplementary material is available at *PNAS Nexus* online.

## Supplementary Material

pgad402_Supplementary_Data

## Data Availability

Cryo-EM structure data have been deposited in the Protein Data Bank (PDB) and Electron Microscopy Data Bank (EMDB). mC-TFG-LCD-G269V—PDB ID: 8TEQ (56); EMDB ID: EMD-41195 (57). mC-TFG-LCD-P285L—PDB ID: 8TER (58); EMDB ID: EMD-41198 (59).
