# Supplementary material for: Fibril structures of TFG protein mutants validate the identification of TFG as a disease-related amyloid protein by the IMPAcT method
Source: PNAS Nexus. 2023 Nov 20;2(12):pgad402. doi: 10.1093/pnasnexus/pgad402 (PMC10703350; doi:10.1093/pnasnexus/pgad402)
Supplement: pgad402_Supplementary_Data [file pgad402_supplementary_data.zip › PNASNEXUS-PNASNEXUS-2023-00974R-s02.pdf]

Supplementary Table 1: IMPAcT results for all variants in the TFG LCD ordered by clinical significance. IMPAcT hits are mutations for which the WT Score is >-23.0 and the Mutant score is <-23.0.

Protein Unirpot ID	WT Score	Mutant Score	WT Residue	Mutant Residue	Position	Gene Name	Clinical Significance
Q92734	-23.36979	-26.240637	A	V	303	TFG	Likely Benign
Q92734	-21.96349	-22.567358	I	T	239	TFG	Likely Benign
Q92734	-18.831463	-20.168476	Q	H	246	TFG	Likely Benign
Q92734	-22.134132	-23.542364	Y	H	276	TFG	Likely Pathogenic
Q92734	-24.405033	-25.273342	G	D	269	TFG	Pathogenic
Q92734	58.122036	-23.774788	P	L	285	TFG	Pathogenic
Q92734	-20.715498	-23.589687	G	V	269	TFG	Pathogenic
Q92734	-27.26337	-27.415707	A	S	326	TFG	VUS
Q92734	-21.794899	-24.510702	Y	C	247	TFG	VUS
Q92734	169.72202	-24.111721	P	S	300	TFG	VUS
Q92734	26.806074	-23.913332	P	L	258	TFG	VUS
Q92734	169.72202	-23.631155	P	T	300	TFG	VUS
Q92734	-22.799578	-22.966446	A	V	274	TFG	VUS
Q92734	-21.90685	-22.152458	G	S	292	TFG	VUS
Q92734	-18.172222	-20.792059	G	S	252	TFG	VUS
Q92734	-19.22684	-19.92206	Q	H	294	TFG	VUS
Q92734	-18.831463	-15.952381	Q	R	248	TFG	VUS
Q92734	-9.860426	-10.650166	Q	H	264	TFG	VUS
Q92734	-9.860426	-10.650166	Q	H	284	TFG	VUS
Q92734	4.2052336	-8.555239	P	L	315	TFG	VUS
Q92734	-8.057104	-8.328894	Q	E	284	TFG	VUS
Q92734	4.2052336	-6.087772	P	S	315	TFG	VUS
Q92734	-7.8464694	-5.255978	Q	R	259	TFG	VUS
Q92734	4.598022	4.962272	Q	R	327	TFG	VUS
