# Supplementary material for: Fibril structures of TFG protein mutants validate the identification of TFG as a disease-related amyloid protein by the IMPAcT method
Source: PNAS Nexus. 2023 Nov 20;2(12):pgad402. doi: 10.1093/pnasnexus/pgad402 (PMC10703350; doi:10.1093/pnasnexus/pgad402)
Supplement: pgad402_Supplementary_Data [file pgad402_supplementary_data.zip › PNASNEXUS-PNASNEXUS-2023-00974R-s01.docx]

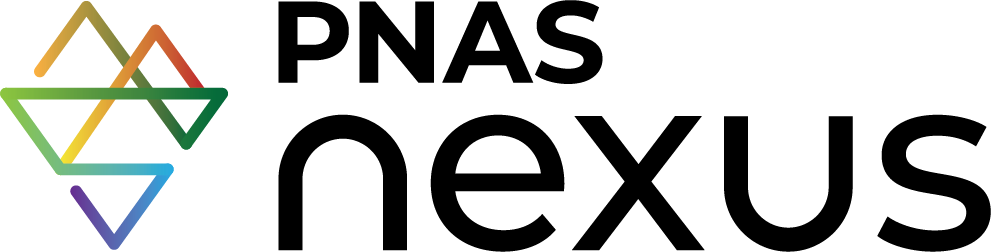


**Supporting Information for**

Fibril structures of TFG protein mutants validate identification of TFG as a disease-related amyloid protein by the IMPAcT method

Gregory M. Rosenberg, Romany Abskharon, David R. Boyer, Peng Ge, Michael R. Sawaya, David S. Eisenberg

David S. Eisenberg

Email: [david@mbi.ucla.edu](mailto:david@mbi.ucla.edu)

**This Word file includes:**

Figures S1 to S8


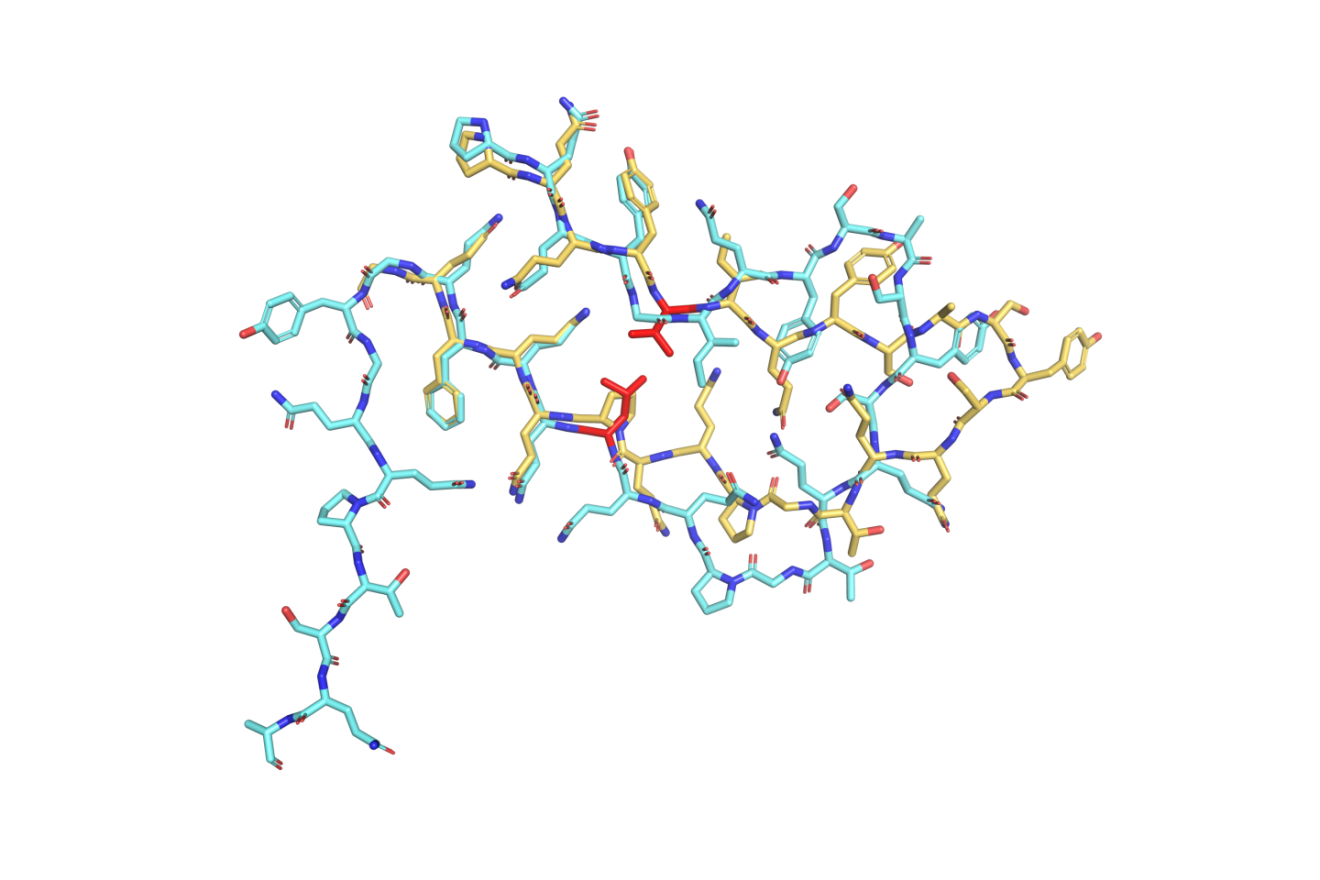


**Fig. S1.** Superposition of the narrow protofilament of the mC-TFG-LCD-G269V fibril (yellow) and one of the protofilaments of the mC-TFG-LCD-P285L fibril (blue) demonstrating the similarity of the glutamine zipper formed by residues Q267, Q287, and Q289 in both structures, but the divergence of the structural similarity past the location of the mutations (mutant residues from each structure are colored red).


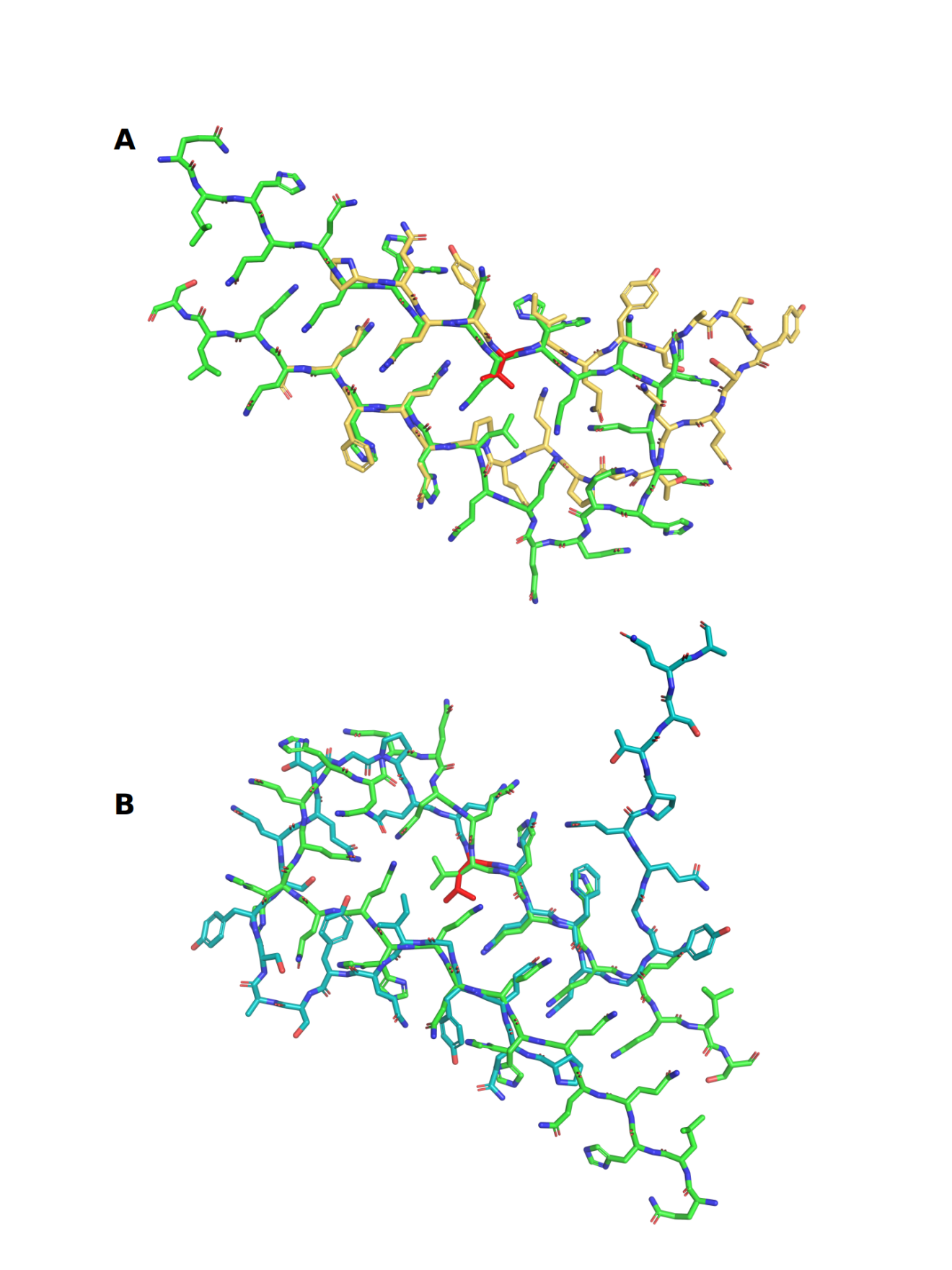


**Fig. S2.** A) Superposition of the narrow filament of the mC-TFG-LCD-G269V fibril (yellow) and one of the protofilaments of the orb2A amyloid fibril (green). B) Superposition of one protofilament of the mC-TFG-LCD-P285L fibril (blue) and one of the protofilaments of the orb2A amyloid fibril (green). Both superpositions are aligned so the glutamine zipper present in both mutant TFG LCD fibril structures (formed by residues Q267, Q287, and Q289) overlaps one of the glutamine zippers of the orb2A fibril structure (in this case residues Q30, Q47, and Q49).

**
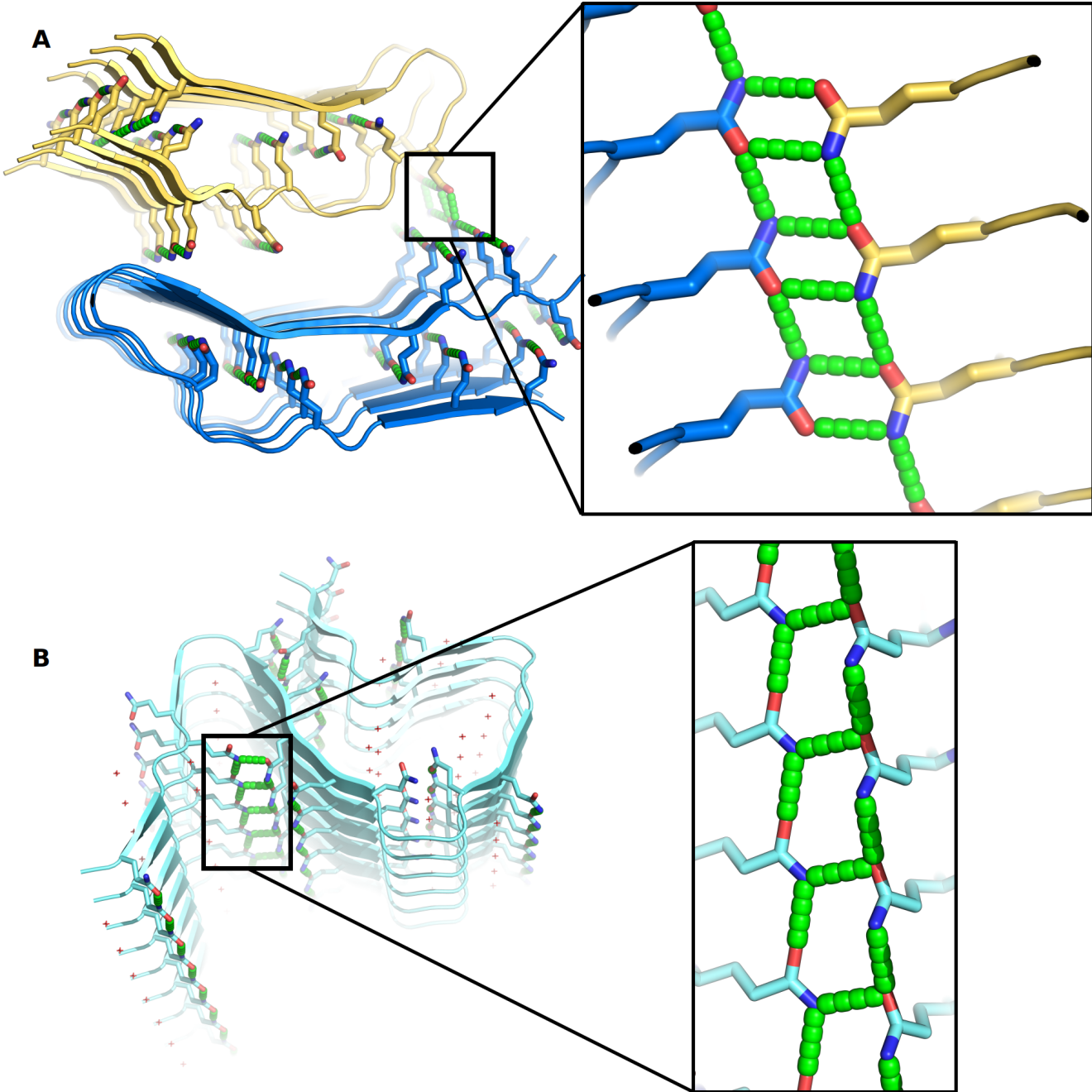
**

**Fig. S3.** A) Cartoon representation of the mC-TFG-LCD-G269V fibril showing only the glutamine side chains which form inter-layer hydrogen bonds (hydrogen bonds are represented by green connections). Inset: glutamine side chains which form inter-protofilament hydrogen bonds as well as inter-layer hydrogen bonds. B) Cartoon representation of one protofilament of the mC-TFG-LCD-P285L fibril showing all glutamine side chains (hydrogen bonds are represented by green connections). Two glutamine side chains are not making interlayer hydrogen bonds: one is facing solvent and one is facing the interior of the protofilament but is solvated by three water molecules. Inset: glutamine side chains which form intra-protofilament hydrogen bonds as well as inter-layer hydrogen bonds.


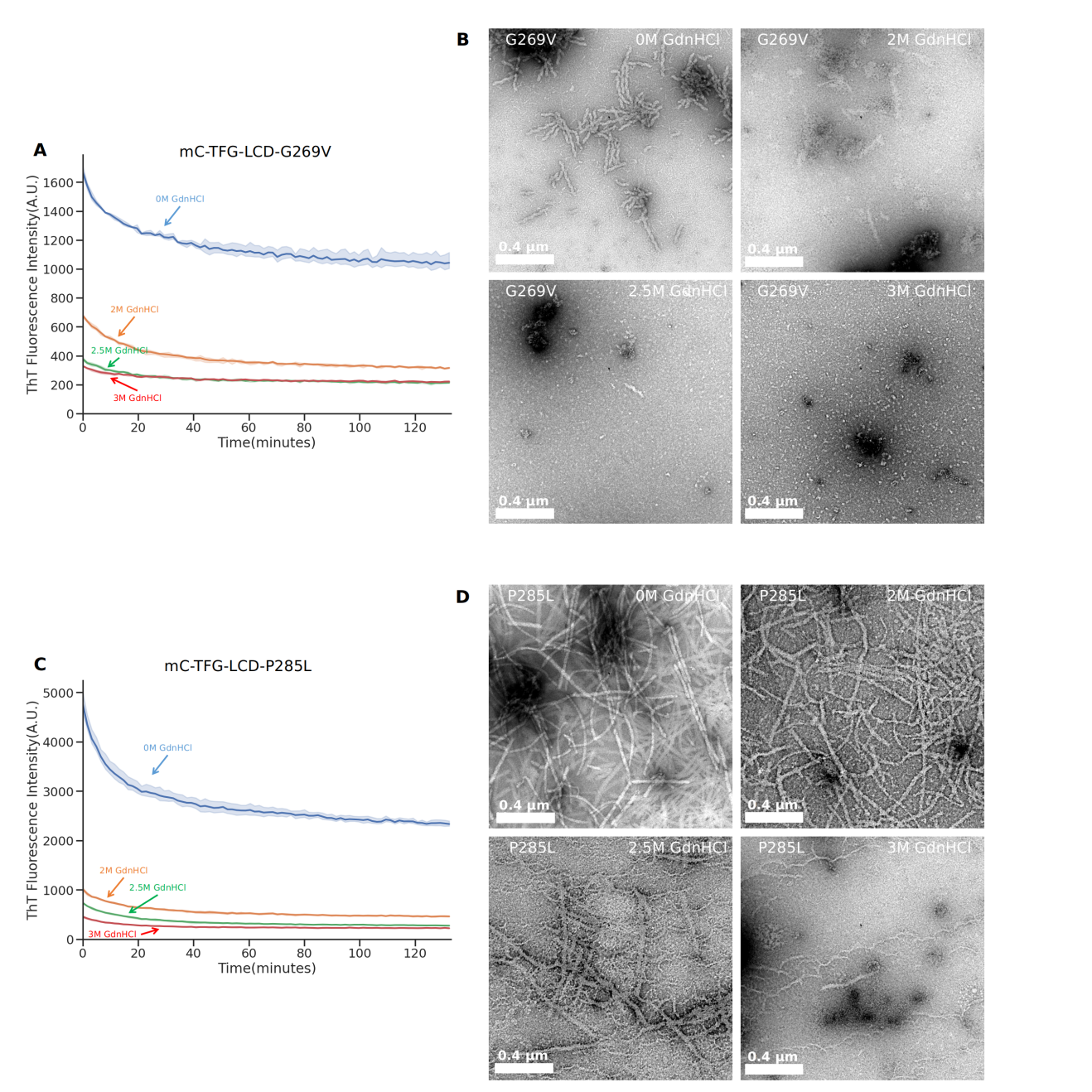


**Fig. S4.** A,C) Time dependent ThT fluorescence of mC-TFG-LCD-G269V fibrils (A) and mC-TFG-LCD-P285L fibrils (C) in solutions of varying concentrations of guanidine hydrochloride (GdnHCl) and 20μM ThT: 0M, 2M, 2.5M, or 3M GdnHCl. Fibrils were generated at 50μM concentration in PBS with ThT at 20μM concentration and diluted 1:5 in the GdnHCl solutions. B,D) Representative TEM images of samples taken from the endpoint of the ThT readings. For the mC-TFG-LCD-G269V fibrils (B), fibrils remained abundant in 2M GdnHCl, but were extremely sparse in 2.5M GdnHCl, and were completely absent in 3M GdnHCl. For the mC-TFG-LCD-P285L fibrils (D), fibrils remained abundant in both 2M and 2.5M GdnHCl, but were extremely sparse in 3M GdnHCl.


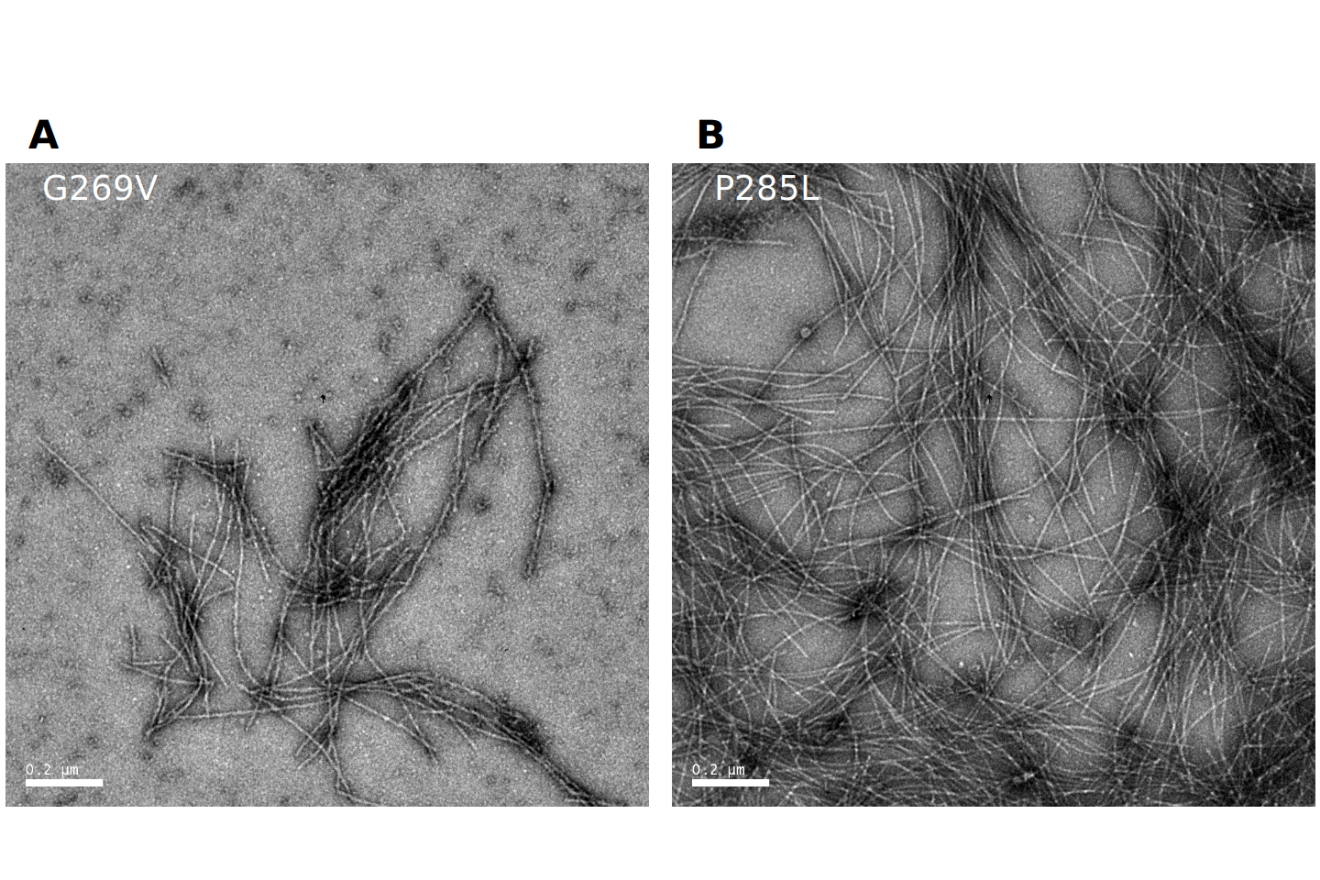


**Fig. S5.** Samples of mC-TFG-LCD-G269V fibrils (A) and mC-TFG-LCD-P285L fibrils (B) which were grown as described in the Methods, diluted 1:5, and stored at 4℃ for approximately 2.5 years.


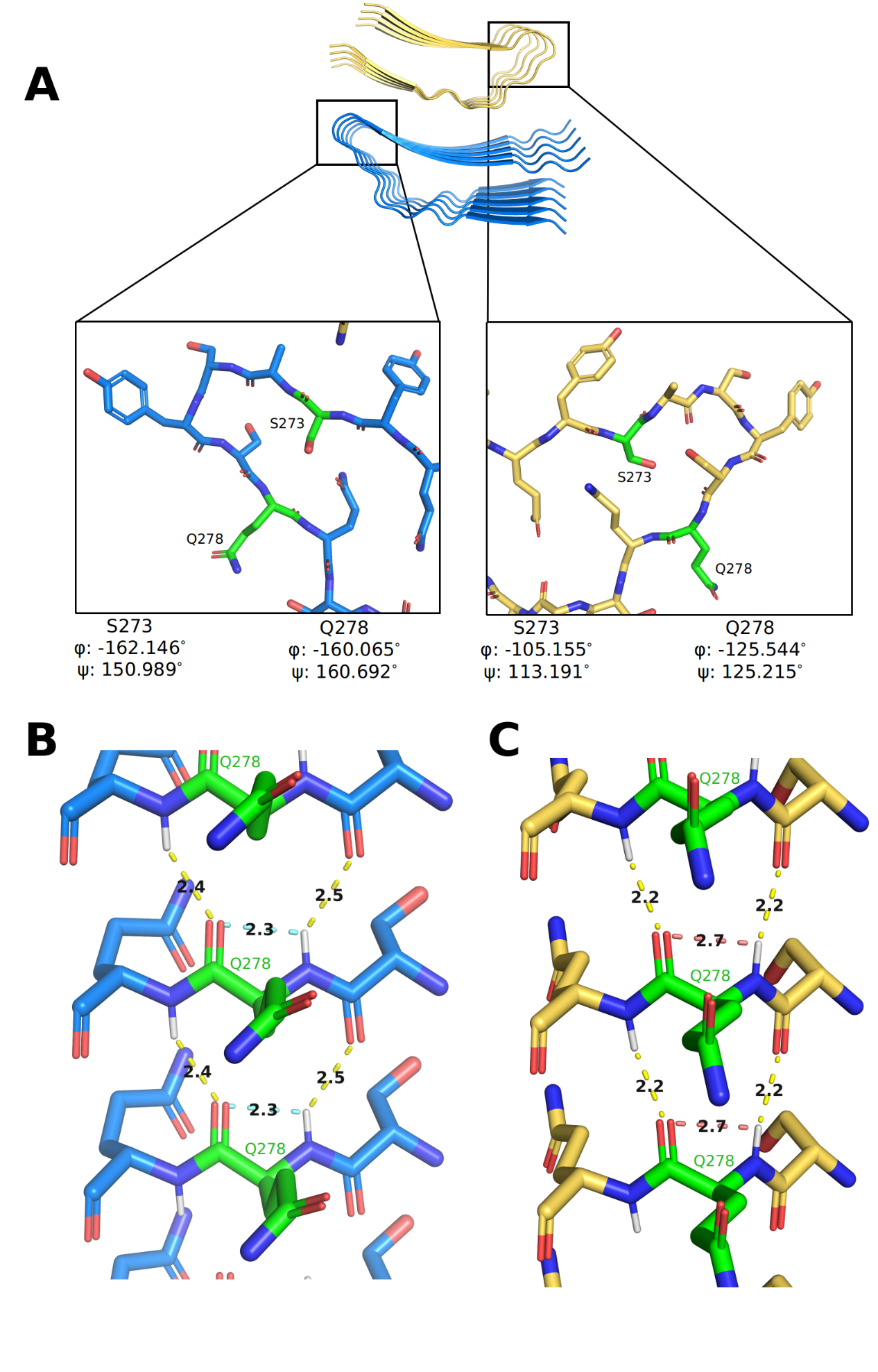


**Fig. S6.** Asymmetric extended β-sheet conformation between mC-TFG-LCD-G269V protofilaments. A) The wide protofilament (blue; inset: left) has two residues (S273 and Q278), colored green, with |φ| and |ψ| angles with values which permit an extended β-sheet conformation (> 150^°^). The narrow protofilament (yellow; inset: right) has no residues with appropriate φ and ψ angles to permit this structure. Residues in the narrow protofilament corresponding to the residues in the wide protofilament with an extended β-sheet conformation are also colored green. An extended β-sheet conformation makes C5-hydrogen bonds possible. φ and ψ angle values for residues S273 and Q278 for each protofilament are listed below each inset of the corresponding protofilament. B,C) Distances between possible hydrogen bond partners of the carbonyl oxygen and amide hydrogen of Q278 in the wide protofilament (B) and the narrow protofilament (C). Distances are represented by dashed lines and labeled with the distance in Angstroms. The cyan dashed line in (B) represents the C5 hydrogen bond conformation where the distance between the oxygen and hydrogen within Q278 is within hydrogen bonding range. The pink dashed line in (C) represents a non-C5 hydrogen bonding conformation where the distance between the oxygen and hydrogen within Q278 is longer than hydrogen bonding range.


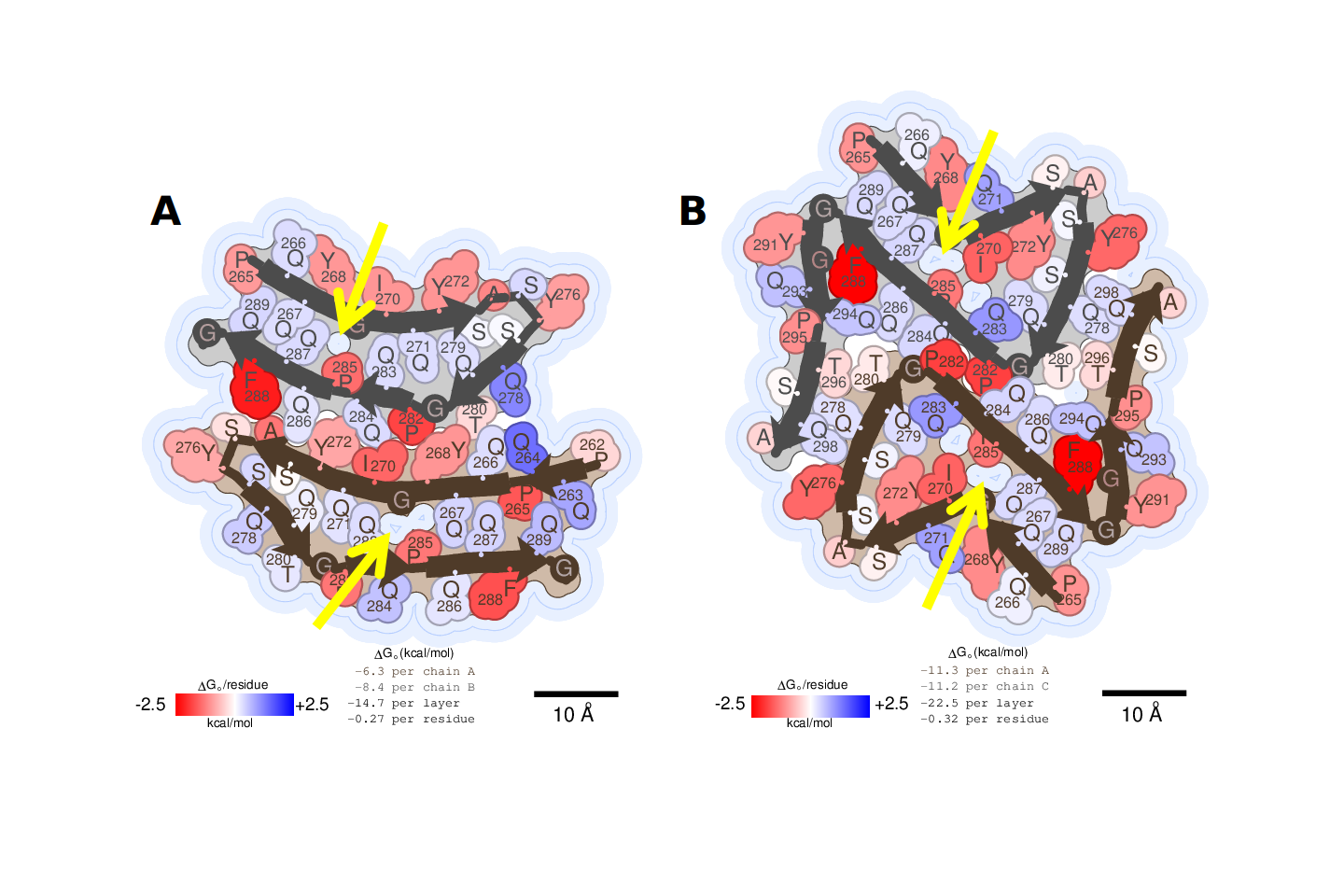


**Fig. S7.** Hypothetical structures of mC-TFG-LCD WT amyloid fibrils. The mutant residues in the TFG LCD fibril structure models were replaced with the WT residues (in the mC-TFG-LCD-G269V model, the mutant valine residue is now the WT glycine; in the mC-TFG-LCD-P285L model, the mutant leucine residue is not the WT proline). A,B) Solvation energy maps of the mC-TFG-LCD-G269V fibril core with the WT sequence (A) and the mC-TFG-LCD-P285L fibril core with the WT sequence (B). Residues are colored according to their stabilization energies. Deeper blue (positive) is unfavorable for amyloid assembly and deeper red (negative) is favorable. The thin dark blue line represents the solvent-accessible surface. Energy values are listed below the structure illustration. In (A) chain A is the wide protofilament and chain B is the narrow protofilament in the illustration. Yellow arrows point to solvent accessible channels which do not exist in the mutant structures.


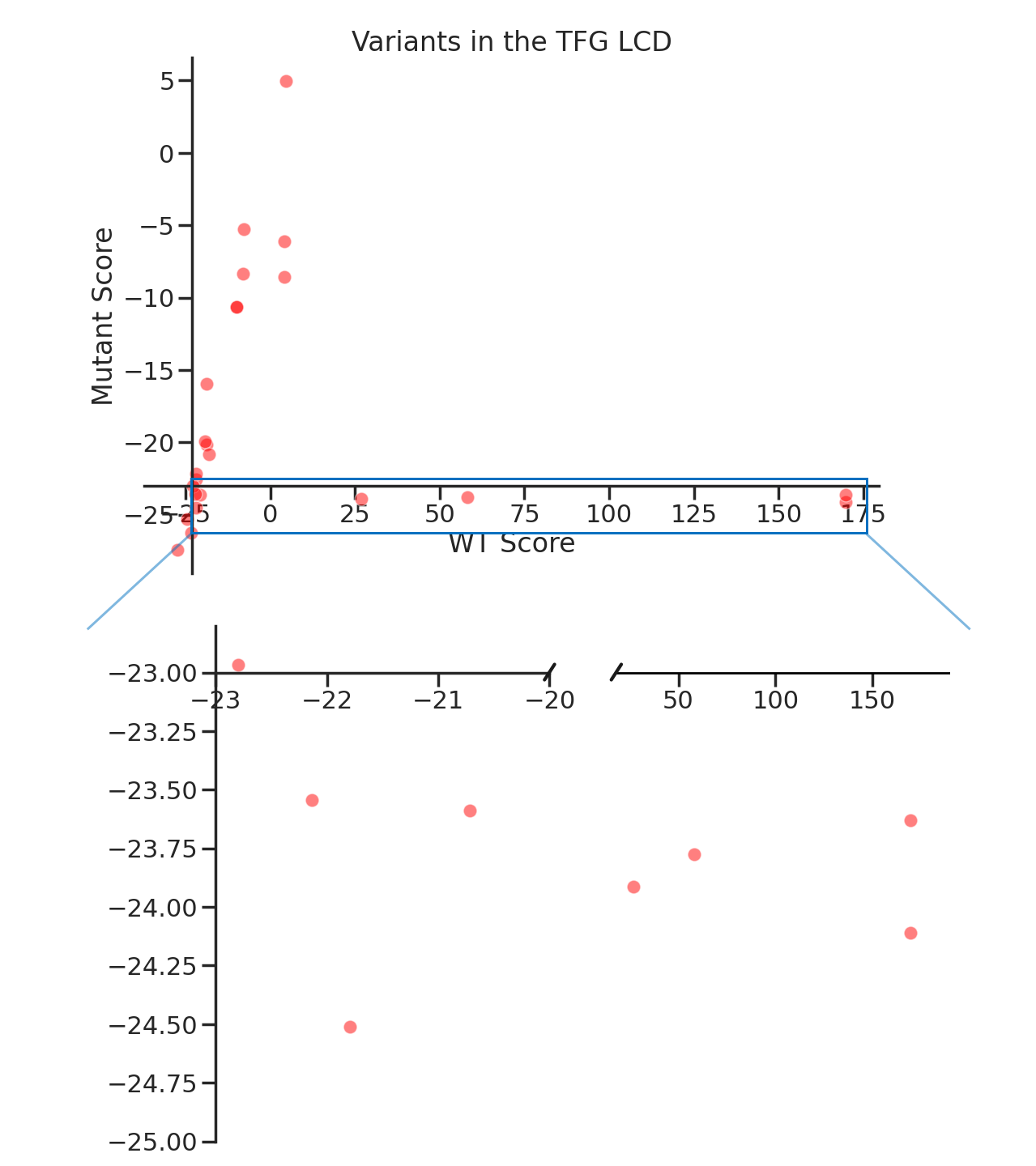


**Fig S8.** Plot of IMPAcT results for 24 variants within the LCD of TFG. The x-axis is the ZipperDB score of the WT sequence and the y-axis is the ZipperDB score of the mutant sequence. Because each mutation generates six possible score pairs, only the score pair that mapped to the “region of interest” (inset) or with the greatest negative change from WT to mutant score is plotted for each mutation. The x and y intercepts are both at −23.0 kcal/mol of the segment, the ZipperDB threshold for a predicted amyloid-forming steric zipper. Inset contains a zoomed view of the lower right quadrant of the plot that is the “region of interest” containing points corresponding to a WT segment with a score above −23.0 kcal/mol of segment and a mutant segment with a score below −23.0 kcal/mol of segment, indicating a mutation that increases the amyloid propensity.
